# Changes of motor corticobulbar projections following different lesion types affecting the central nervous system in adult macaque monkeys

**DOI:** 10.1111/ejn.14074

**Published:** 2018-08-16

**Authors:** Michela Fregosi, Alessandro Contestabile, Simon Badoud, Simon Borgognon, Jérôme Cottet, Jean‐François Brunet, Jocelyne Bloch, Martin E. Schwab, Eric M. Rouiller

**Affiliations:** ^1^ Faculty of Science and Medicine Section of Medicine Department of Neurosciences and Movement Sciences University of Fribourg Fribourg Switzerland; ^2^ Fribourg Cognition Center Fribourg Switzerland; ^3^ Platform of Translational Neurosciences Fribourg Switzerland; ^4^ Swiss Primate Competence Center for Research (SPCCR) Fribourg Switzerland; ^5^ Cell production center (CPC) Lausanne University Hospital (CHUV) Lausanne Switzerland; ^6^ Department of Neurosurgery Lausanne University Hospital (CHUV) Lausanne Switzerland; ^7^ Brain Research Institute University of Zürich Zürich Switzerland

**Keywords:** anterograde tracing, brainstem, cortical lesion, motor cortex, nonhuman primate, Parkinson, spinal cord injury

## Abstract

Functional recovery from central nervous system injury is likely to be partly due to a rearrangement of neural circuits. In this context, the corticobulbar (corticoreticular) motor projections onto different nuclei of the ponto‐medullary reticular formation (PMRF) were investigated in 13 adult macaque monkeys after either, primary motor cortex injury (MCI) in the hand area, or spinal cord injury (SCI) or Parkinson's disease‐like lesions of the nigro‐striatal dopaminergic system (PD). A subgroup of animals in both MCI and SCI groups was treated with neurite growth promoting anti‐Nogo‐A antibodies, whereas all PD animals were treated with autologous neural cell ecosystems (ANCE). The anterograde tracer BDA was injected either in the premotor cortex (PM) or in the primary motor cortex (M1) to label and quantify corticobulbar axonal boutons *terminaux* and *en passant* in PMRF. As compared to intact animals, after MCI the density of corticobulbar projections from PM was strongly reduced but maintained their laterality dominance (ipsilateral), both in the presence or absence of anti‐Nogo‐A antibody treatment. In contrast, the density of corticobulbar projections from M1 was increased following opposite hemi‐section of the cervical cord (at C7 level) and anti‐Nogo‐A antibody treatment, with maintenance of contralateral laterality bias. In PD monkeys, the density of corticobulbar projections from PM was strongly reduced, as well as that from M1, but to a lesser extent. In conclusion, the densities of corticobulbar projections from PM or M1 were affected in a variable manner, depending on the type of lesion/pathology and the treatment aimed to enhance functional recovery.

**Abbreviations**
BDA: biotinylated dextran amine
CM: corticomotoneuronal
CNS: central nervous system
CS: corticospinal
CST: corticospinal tract
DA: dopamine
Gi: gigantocellularis reticular nucleus
IRt: intermediate reticular nucleus
LRt: lateral reticular nucleus
M1: primary motor cortex
MPTP: 1‐methyl‐4phenyl‐1,2,3,6‐tetrahydropyridine
PD: Parkinson's disease
PMd: dorsal premotor cortex
PM: premotor cortex
PMRF: ponto‐medullary reticular formation
PMv: ventral premotor cortex
PnC: pontine reticular nucleus pars caudalis
PnO: pontine reticular nucleus pars oralis
Pn: pontine nuclei
RS: reticulospinal
RST: reticulospinal tract
Rt: reticular nuclei
SMA: supplementary motor area

## 1 INTRODUCTION

The motor corticobulbar (corticoreticular) projections, acting in parallel with the corticospinal (CS) tract in the control of voluntary movements, terminate in brainstem nuclei, from which subsequent descending pathways arise to reach the spinal cord; one of these is the reticulospinal tract (RS; Kuypers, 1958, 1981; Lemon, 2008). The RS projection originates from the ponto‐medullary reticular formation (PMRF; Kuypers, 1981; Matsuyama, Takakusaki, Nakajima, & Mori, 1997; Matsuyama, Mori, Kuze, & Mori, 1999; Sakai, Davidson, & Buford, 2009; Fregosi, Contestabile, Hamadjida, & Rouiller, 2017), a portion of the brainstem comprising several reticular nuclei: the pontine reticular nucleus *pars oralis* (PnO) and *pars caudalis* (PnC) as well as the gigantocellular reticular nucleus (Gi) (Kuypers, 1981; Sakai et al., 2009). The RS projection is involved in the control of posture and locomotion (Drew, Dubuc, & Rossignol, 1986; Lawrence & Kuypers, 1968a,b; Matsuyama & Drew, 1997; Matsuyama et al., 1999, 2004; Schepens & Drew, 2004, 2006; Schepens, Stapley, & Drew, 2008), as well as in the control of reaching movements (Buford & Davidson, 2004; Davidson & Buford, 2004, 2006; Davidson, Schieber, & Buford, 2007; Schepens & Drew, 2004, 2006; Schepens et al., 2008). More recently, evidence was provided that the RS projection is also involved in the control of hand movements, via monosynaptic or disynaptic connections with motoneurons controlling intrinsic hand muscles (Baker, 2011; Riddle & Baker, 2010; Riddle, Edgley, & Baker, 2009; Soteropoulos, Williams, & Baker, 2012). The relationship between the RS projection and hand movements has been extended to humans (Honeycutt, Kharouta, & Perreault, 2013).

Besides the role played by the RS projection in the control of hand movement, the main player for hand control remains the corticospinal tract (CST) mainly via its corticomotoneuronal (CM) system allowing sophisticated control of manual dexterity in nonhuman primates and humans (Courtine et al., 2007; Lawrence & Kuypers, 1968a,b; Lemon, 2008; Lemon & Griffiths, 2005; Rathelot & Strick, 2009; Schieber, 2007). Rathelot and Strick (2009) demonstrated that M1 can be subdivided into an “old” M1 and a “new” M1. The former is the rostral region of M1 and connects to spinal cord motoneurons only disynaptically, whereas the latter corresponds to the caudal region of M1 and contains almost all CM neurons connecting directly to spinal cord motoneurons. In both primates and rodents the CS projection sends bilateral projections (though mostly crossed) (Fink & Cafferty, 2016; Lacroix et al., 2004; Lemon, 2008; Rosenzweig et al., 2009). The motor system shows some functional redundancy between its multiple descending motor pathways, which may allow intact pathways to rearrange and support functional recovery following a lesion of one of them (e.g. Fink & Cafferty, 2016; Galea & Darian‐Smith, 1997; Herbert, Powell, & Buford, 2015; Lemon, 2008; Zaaimi, Edgley, Soteropoulos, & Baker, 2012).

Damage to the CS system due either to stroke (affecting the hand area of the motor cortex) or to cervical spinal cord lesion, causes impairments of the manual dexterity and flaccid paralysis in a first step (Freund et al., 2006, 2007, 2009; Galea & Darian‐Smith, 1997; Kaeser et al., 2010, 2011; Lawrence & Kuypers, 1968a,b; Lemon, 2008; Liu & Rouiller, 1999; Wannier, Schmidlin, Bloch, & Rouiller, 2005). Parkinson's disease (PD), caused by a dopamine depletion in the striatum of the projection originating from the substantia nigra pars compacta, is characterized by motor symptoms such as tremors, bradykinesia, rigidity and postural instability, when the DA loss reaches about 70%–80% or more (e.g. Emborg, 2007; Fitzpatrick, Raschke, & Emborg, 2009). To the best of our knowledge, the issue of how the motor corticobulbar projections are modified following one of these three pathologies (motor cortex lesion, cervical cord injury or PD) has not been investigated in nonhuman primates. To do so, the present report is derived from previous behavioural experiments in three cohorts of macaques, which were subjected either to lesion of the primary motor cortex (MCI = motor cortex injury), lateral hemi‐section of the cervical cord (SCI = spinal cord injury), or 1‐methyl‐4phenyl‐1,2,3,6‐tetrahydropyridine (MPTP) intoxication to produce Parkinson's disease‐like lesions of the nigro‐striatal dopaminergic system (PD). Previous reports derived from these experiments concerned specifically behavioural data (Badoud et al., 2017; Bashir et al., 2012; Freund et al., 2006, 2009; Hamadjida et al., 2012; Hoogewoud et al., 2013; Kaeser et al., 2010, 2011; Wyss et al., 2013), imaging of dopaminergic function (Borgognon et al., 2017) or tracing data on the CS tract (Beaud et al., 2008; Freund et al., 2007), the rubrospinal projection (Wannier‐Morino et al., 2008) or callosal projection (Hamadjida et al., 2012). In the present study, this rich collection of monkeys subjected to one of these pathologies (MCI, SCI, PD; *n* = 13) was analysed here in order to compare their corticobulbar projections to PMRF with that of a cohort of intact monkeys (*n* = 7) recently reported (Fregosi et al., 2017). In addition, it was our goal to investigate whether a treatment aiming at neutralizing the neurite growth inhibitor Nogo‐A (anti‐Nogo‐A antibody treatment: see Freund et al., 2006, 2009; Hamadjida et al., 2012; Wyss et al., 2013) interferes with the plasticity of the corticobulbar projection following MCI or SCI. Finally, the four PD macaques were subjected to an autologous cell therapy (see Bloch, Brunet, McEntire, & Redmond, 2014; Brunet, Redmond, & Bloch, 2009; Brunet, Rouiller, Wannier, Villemure, & Bloch, 2005; Kaeser et al., 2011). In summary, the present study tested the hypothesis that the motor corticobulbar projections to PMRF are significantly modified following MCI or SCI or PD and that anti‐Nogo‐A antibody treatment interferes with these lesion‐induced plastic changes.

## 2 MATERIALS AND METHODS

The corticobulbar projections arising from either PM or M1 in the PMRF of 13 macaque monkeys (*Macaca fascicularis*, seven males and six females, aged 4–9.5 years at sacrifice, weighing 3.3–5.6 kg either at sacrifice or at lesion) were analysed (Table 1).

**Table 1:** Individual monkeys’ data

	Cortical lesion (MCI)^a^				Parkinsonian lesion^b^				Spinal cord lesion^c^				
	Mk‐MO	Mk‐VA	Mk‐RO	Mk‐BI	Mk‐LY	Mk‐MI	Mk‐LL	Mk‐MY	Mk‐CG	Mk‐CP	Mk‐AC	Mk‐AP	Mk‐AG
Species	M.fasc.	M.fasc.	M.fasc.	M.fasc.	M.fasc.	M.fasc.	M.fasc.	M.fasc.	M.fasc.	M.fasc.	M.fasc.	M.fasc.	M.fasc.
Sex	Male	Male	Male	Male	Female	Female	Female	Female	Male	Female	Male	Female	Male
Age sacrifice (rounded 0.5 years)	6	6	4.5	6	7.5	9.5	7.5	9.5	4	7	4	6.5	3.5
Age lesion (rounded 0.5 years)	5.5	5.5	4	5	7	9	7	9	3.5	6.5	3.5	6	3
Weight (sacrifice or lesion)	5.6	4.9	3.2	5	3.3	3.3	3.6	4.3	*	*	*	*	*
Lesion Type/Location	MCI	MCI	MCI	MCI	PD	PD	PD	PD	SCI	SCI	SCI	SCI	SCI
Lesioned hemisph./SC side	Left	Left	Left	Left	Both	Both	Both	Both	Left	Left	Left	Left	Left
Type of lesion	Ibo. acid	Ibo. acid	Ibo. acid	Ibo. acid	MPTP	MPTP	MPTP	MPTP	Hemi‐sec	Hemi‐sec	Hemi‐sec	Hemi‐sec	Hemi‐sec
Treatment	a‐NogoA	a‐NogoA	‐	‐	ANCE	ANCE	ANCE	ANCE	Control	Control	a‐NogoA	a‐NogoA	a‐NogoA
Tracer injected	BDA	BDA	BDA	BDA	BDA	BDA	BDA	BDA	BDA	BDA	BDA	BDA	BDA
Side injected with BDA	Left	Left	Left	Left	Right	Right	Right	Left	Right	Right	Right	Right	Right
BDA injection sites in:	PMd/PMv	PMd/PMv	PMd	PMd/PMv	M1	M1	PMd/PMv	PMd/PMv	M1	M1	M1	M1	M1
BDA‐injected volume (μl)	10.8	5	4.8	7.2	9	9	9.7	11.5	24	24	20	24	28
Number of BDA inj. sites	12	5	6	11	6	6	8	9	12	12	10	12	15
Number of labelled CST axons	1975	1312	543	1328	1671	1117	593	611	716	712	537	1024	1405
Total volume of lesion (mm^3^) in the grey matter	41.8	20	14	20.1	‐	‐	‐	‐	‐	‐	‐	‐	‐
Number of ibo. acid sites	20	11	12	29	‐	‐	‐	‐	‐	‐	‐	‐	‐
Volume ibo. acid (μl) or MPTP (mg/kg)	20	15.5	18	29.7	6.25	7.75	6.25	6.25	‐	‐	‐	‐	‐
Volume lesion in postcentral gyrus (mm^3^)	0	5.8	0	0	‐	‐	‐	‐	‐	‐	‐	‐	‐
Number of ANCE sites	‐	‐	‐	‐	6 (3/hr)	6 (3/hr)	6 (3/hr)	6 (3/hr)	‐	‐	‐	‐	‐
Loss DA cells in SNpc (%)	‐	‐	‐	‐	−39%	−74%	−67%	−72%	‐	‐	‐	‐	‐
% decrease of 18F‐DOPA uptake in striatum post‐MPTP lesion^d^	‐	‐	‐	‐	−17%	−79%	−84%	−81%	‐	‐	‐	‐	‐
% decrease of 18F‐DOPA uptake in striatum post‐ANCE transplantation^d^					0	−68%	−70%	−60%					
Volume spinal lesion (mm^3^)	‐	‐	‐	‐	‐	‐	‐	‐	1.802	1.782	4.577	1.348	e
Extent hemi‐cord lesion (%)	‐	‐	‐	‐	‐	‐	‐	‐	51	45	85	58	78
Extent of grey matter cut (%)	‐	‐	‐	‐	‐	‐	‐	‐	73	77	100	85	81

Note

The number of labelled corticospinal tract (CST) axons was used in each monkey to normalize the boutons data (see methods).

MCI, motor cortex injury (M1 hand representation unilaterally); PD, Parkinson's disease‐like lesions of the nigro‐striatal dopaminergic system; SCI, spinal cord injury (Hemi‐section at C7 level); a‐NogoA, treatment with anti‐Nogo‐A antibody; the monkeys Mk‐CG and Mk‐CP received a control antibody; the monkeys Mk‐RO and Mk‐BI were untreated; ANCE, autologous neural cell ecosystems (autologous adult neural progenitor cells reimplanted bilaterally in caudate nucleus and putamen, three sites per hemisphere); Ibo‐acid, ibotenic acid (with indication of the number of sites where it was injected in M1).

*Data not available.

^a^Derived from previous behavioural studies: Kaeser et al. (2010, 2011); Bashir et al. (2012); Hamadjida et al. (2012); Hoogewoud et al. (2013); Wyss et al. (2013). ^b^Derived from previous behavioural studies: S. Badoud (Ph.D. thesis 2016); Badoud et al. (2017); Borgognon et al. (2017). ^c^Derived from previous behavioural studies: Freund et al. (2006, 2007, 2009); Wannier‐Morino et al. (2008); Beaud et al. (2008); Hoogewoud et al. (2013). ^d^Percentage decrease with reference to 18F‐Dopa uptake in striatum prelesion. It means that 18F‐Dopa uptake reincreased post‐ANCE transplantation as compared to post‐MPTP lesion, as recently reported (Borgognon et al., 2017). ^e^Lesion volume in Mk‐AG could not be determined due to a few missing histological sections.

The monkeys were purpose bred for research in duly authourized breeding colonies in Asia, then imported to a quarantine centre (also duly authorized) in Europe, where the health status of the animals was checked and complemented (tests, vaccines, etc.) to fulfil legal requirements before final transfer to Switzerland, with the authorization of the Federal Veterinary Office.

The animals were involved in one out of three different lesion/pathology types: four animals (Mk‐MO, Mk‐VA, Mk‐RO and Mk‐BI) were subjected to cortical lesion of the hand area of M1 (MCI), four animals (Mk‐LL, Mk‐MY, Mk‐MI and Mk‐LY) were subjected to MPTP injections mimicking PD and finally five animals (Mk‐CG, Mk‐CP, Mk‐AC, Mk‐AP and Mk‐AG) were subjected to cervical cord hemi‐section (SCI). The MCI and SCI monkeys were housed in groups of 2–5 animals in detention rooms of 15 m^3^, according to guidelines valid until 2010. The MPTP monkeys were housed after 2010 in groups of 2–5 animals in a detention room of 45 m^3^ (new guidelines). The number of monkeys was minimized as much as possible by limiting the number of cases per lesion/pathology and using the same cases to trace other pathways as well (e.g. Beaud et al., 2012; Freund et al., 2007; Hamadjida et al., 2012). The authors adhere to the NC3Rs initiative (“replace, reduce, refine” the use of animals in research; http://www.nc3rs.org.uk) and are members of the Basel Declaration (http://www.basel-declaration.org).

All 13 animals were also involved in behavioural tasks, as previously reported (see Badoud et al., 2017; Bashir et al., 2012; Freund et al., 2006, 2009; Hamadjida et al., 2012; Hoogewoud et al., 2013; Kaeser et al., 2010, 2011; Schmidlin et al., 2011; Wyss et al., 2013). All surgical and experimental procedures, animal care were conducted in accordance to the Guide and Care and Use of Laboratory animals (ISBN 0‐309‐05377‐3, 1996) and were authorized by both the local (Canton of Fribourg) and federal (Switzerland) veterinary offices (authorizations FR‐157‐03; FR‐157‐04; FR‐157e‐04; FR‐156‐04; FR‐156‐06; FR‐185‐08; FR‐17‐09; FR‐2012‐01; FR‐2012‐01E). All precautions and measures were taken to minimize pain and constraints to the animals (e.g. analgesia postsurgical interventions, antibiotics, etc.). For comparison of the corticobulbar projection, the data derived from these 13 lesioned monkeys were compared to data obtained in seven intact animals (Fregosi et al., 2017: see their Table 1 for individual data of the intact monkeys).

### 2.1 Surgical procedures

**Figure 1:**
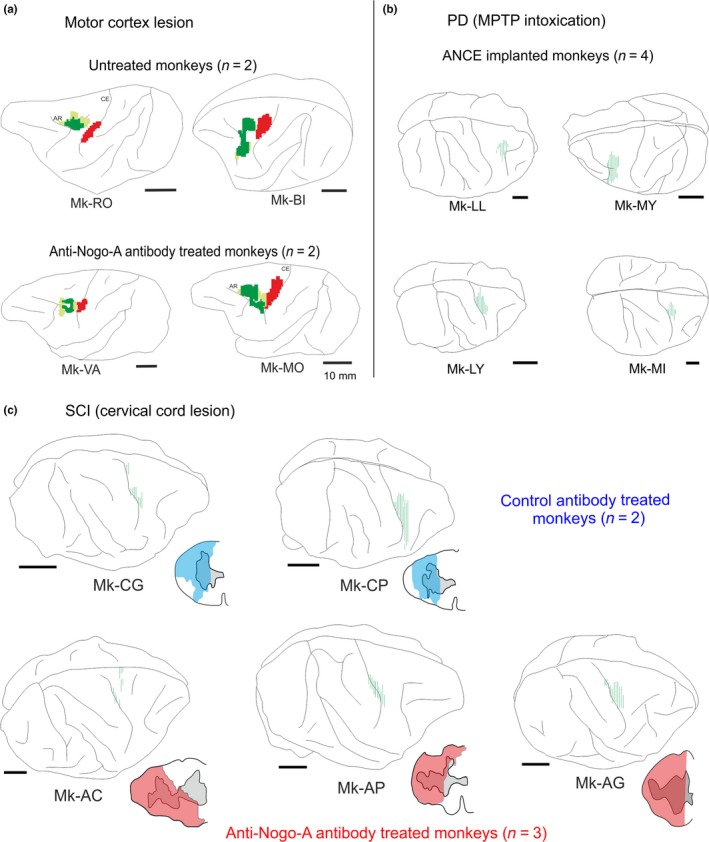
(a) On lateral view of the left hemisphere, reconstruction of the BDA injection site in PM (green) and of the M1 lesion (red) for the MCI monkeys (data derived from Hamadjida et al., 2012). For these PM injections, in addition to a dense core region (dark green), an additional halo part was visible where a less dense BDA spread was present. (b) Lateral view of the hemisphere in PD animals with BDA injection sites (green) in PM (top two monkeys) and in M1 (bottom two monkeys). (c) Lateral view of the right hemisphere in SCI monkeys with BDA injection sites (green) in M1. Next to each hemisphere, an inset illustrates the cervical cord lesion (blue or red area) in the same monkey, as seen on a frontal section of the cervical cord (derived from Freund et al., 2007; Beaud et al., 2008, 2012). In each panel (a, b, c), the treatment applied to each animal is indicated

The four cortically lesioned animals (MCI: Mk‐MO, Mk‐VA, Mk‐RO and Mk‐BI) were deeply anesthetised as previously reported (Schmidlin et al., 2005; Freund et al., 2006; Wannier et al., 2005) and chronic chambers were implanted for easier access to the hand region of M1. Cortical lesion of M1 hand area was obtained by multiple injections of ibotenic acid (10 μg/μl in phosphate buffered saline) with a 10‐μl Hamilton syringe at multiple sites corresponding to digits areas previously identified by ICMS, as previously reported (Hamadjida et al., 2012; Kaeser et al., 2010, 2011; Liu & Rouiller, 1999; Wyss et al., 2013). Detailed information about the cortical lesion volume and location is available in Table 1 and Figure 1.

Cervical hemi‐spinal cord lesion at C7 level (SCI) was performed with a surgical blade and all the protocols used for sedation, surgery procedures as well as postoperative cares were previously published (see Freund et al., 2006, 2007, 2009; Wannier et al., 2005). Information about the cervical cord lesion size and location is given in Table 1 and Figure 1.

Animals involved in Parkinsonian lesion (PD: Mk‐LY, Mk‐LL, Mk‐MY and Mk‐MI; see Borgognon et al., 2017) underwent daily MPTP, 0.5 mg/Kg, Sigma; i.m. injections (see Mounayar et al., 2007) with the purpose to destroy dopaminergic neurons in the substantia nigra *pars compacta* (SNpc). During the lesion period (about 1 month), the animals were transferred daily in small cages with a movable Plexiglas plate in order to reduce the risks for the experimenter and to get an easier access to the targeted muscles. Once injected, the animal was placed back in its home cage. At the end of the lesional protocol the animals had received between 6.25 mk/kg and 7.75 mg/kg of MPTP (Table 1; see also Borgognon et al., 2017). The protocol consisted of two series of injections for 4 days each with a break after the fourth day. After the second break the animals received between four and five extra injections (0.25 mg/Kg) depending on their symptoms. The symptoms were assessed according to the Schneider rating scale (Schneider, Lidsky, Hawks, Mazziotta, & Hoffman, 1995). All safety protocols were adapted in accordance to the American national institute of health (NIH) and were based on a previously published technical report of Przedborski et al. (2001). Information about dopaminergic neuronal loss in SNpc, as well as about decrease of ^18^F‐DOPA uptake in the striatum (post‐MPTP lesion and post‐ANCE transplantation), is available in Table 1 (see also Borgognon et al., 2017).

### 2.2 Treatments

All animals involved in the present study were subjected to a “therapeutic” treatment with exception of Mk‐RO and Mk‐BI (untreated monkeys), which were both subjected to M1 lesion (Table 1). The other two cortically lesioned animals (Mk‐MO and Mk‐VA) were treated with anti‐Nogo‐A antibodies (3 mg/ml) administrated by two osmotic pumps (Alzet, model 2ML2, 5 μl/hr) implanted under deep anaesthesia in a subcutaneous pouch in the neck region. One pump administered the treatment intrathecally to the cervical spinal cord (see Freund et al., 2006, 2007, 2009), whereas the other pump delivered the antibody close to the lesioned site in M1 below the dura. The treatment was delivered for a total duration of 4 weeks before surgical removal of the two osmotic pumps. The treatment was delivered immediately after the lesion, thus the surgery for pumps implantation followed the injection of ibotenic acid (Hamadjida et al., 2012; Wyss et al., 2013).

All five animals subjected to SCI were treated with antibodies applied intrathecally by infusion over 4 weeks into the subdural space of the lower cervical spinal cord (Table 1). Mk‐CG and Mk‐CP received a control antibody, whereas Mk‐AC, Mk‐AG and Mk‐AP received anti‐Nogo‐A antibodies (see Beaud et al., 2008; Freund et al., 2006, 2007). In both cases, the treatment was delivered with an osmotic pump (Alzet, 2ML2, 2 ml of volume) located in the back of the animal at 3–5 mm rostral to the lesion site and connected to it by a silastic tube. The implantation of the osmotic pump was performed a few minutes after the cervical lesion itself (Freund et al., 2006, 2007).

Parkinson's disease animals were treated with autologous neural cell ecosystems (ANCE: see Borgognon et al., 2017) obtained by cell culture of cortical neurons developed in vitro from cortical biopsies (Table 1). The protocol for cortical biopsy surgery was the same as that previously published (Badoud et al., 2017), whereas the protocols to prepare the cell culture and cell labelling were the same as previously used (Bloch et al., 2014; Brunet et al., 2005, 2009). ANCE were then reimplanted in the same monkey (sedation, surgical procedure and postimplantation treatment was the same as that used for cortical biopsies). The injections sites (two in the Putamen and one in the Caudate nucleus on each side) were identified by aid of postlesional T1‐weighted MRI scan (OsiriX, v 4.1.2) and confirmed by the Paxinos atlas (Paxinos, Huang, & Toga, 2000). Cells were implanted via a Hamilton microsyringe (10 μl culture medium; 2 μl/min during 5 min per injection site) using a nanoinjector (Stoelting, Wood Dale, IL, USA) fixed to a stereotaxic frame. Each animal received between 250,000 and 400,000 cells (Borgognon et al., 2017).

### 2.3 Sacrifice

At the end of the experimental protocol, after completion of behavioural and electrophysiological investigations, we injected the anterograde tracer biotinylated dextran amine (BDA) in either PM (*n* = 4 cortically lesioned animals and *n* = 2 PD animals, namely Mk‐LL and MK‐MY) or in M1 (*n* = 5 spinal cord injured monkeys and *n* = 2 PD animals, namely MK‐LY and Mk‐MI). The volume of BDA injected, the number of injected sites, the hemisphere injected and the number of CS axons labelled (used as a reference for normalization) are indicated in Table 1. Following a survival time of 3 weeks to allow the axonal transport of BDA (down to the cervical cord), the monkeys were euthanized. Surgical procedure for sacrifice in cortical lesioned animals (*n* = 4) and spinal cord injured animals (*n* = 5) were previously reported (Hamadjida et al., 2012; Wannier et al., 2005; Wyss et al., 2013). In the four animals subjected to PD lesion the euthanasia protocol was different. They were sedated by i.m injection of ketamine (10 mg/Kg) before an i.v. injection of sodium pentobarbital (60 mg/Kg). Once deep anaesthesia was present and the heart rate significantly dropped (as assessed with ECG), 1 ml of heparin was injected in the left ventricle and then the animal was subjected to transcardiac perfusion (400 ml, 0.9% saline). The animals were further perfused with first 3 L 4% paraformaldehyde (0.1 M phosphate buffer, pH 7.6) and then three series of 2 L each of sucrose solutions (10%, 20% and 30%) to fix and cryopreserve the tissue.

### 2.4 Histology, data analysis and statistics

The procedures and the analysis performed in PMRF on brainstem tissue (series stained one with BDA and one with Nissl) are the same as those recently published (Fregosi & Rouiller, 2017; Fregosi et al., 2017) for intact monkeys. Briefly, the relevant extent of the brainstem comprising PMRF included usually 12 sections from an individual series of sections, which were scanned using an Olympus BX40 microscope interfaced with the Neurolucida^®^ software (MBF, Bioscience‐MicroBrightField, Inc. Version 11). The interval between consecutive analysed sections ranged from 0.9 to 1 mm. The Nissl series was used to delineate the nuclei, and then they were aligned to the corresponding BDA adjacent sections used to chart the axonal boutons (*en passant* and *terminaux*) and stem axons. Boutons *en passant* were defined as boutons along an axon segment, visible at magnification 200x, exhibiting a diameter at least twice that of the axon's diameter. In each analysed BDA histological section, all labelled corticobulbar axonal boutons (*en passant* and *terminaux*) were counted in the delineated area of the brainstem, corresponding to a quantification method based on exhaustive plotting instead of stereology (see Fregosi & Rouiller, 2017). Furthermore, across monkeys, all histological samples were analysed according to the same procedure.

The size and precise location of the BDA injection sites is inevitably variable across monkeys, and therefore this may impact the uptake of BDA by corticobulbar neurons in the cerebral cortex, namely in layer V where they are located. In order to tentatively normalize the number of corticobulbar boutons in the brainstem, the number of CS axons labelled with BDA was assessed and taken as a reference. In each monkey, a frontal section of the brainstem taken just above the pyramidal decussation was used to count the number of BDA labelled CS axons in the pyramid. In a previous study (Rouiller, Moret, Tanné, & Boussaoud, 1996), it was found that such counts repeated on consecutive sections in the same animal yielded comparable numbers and therefore, in most subsequent cases, the count of CS axons was performed on a single brainstem section. For normalization, the absolute number of corticobulbar boutons was divided by the total number of BDA‐labelled CS axons, then multiplied by 1000, corresponding to the normalized number of corticobulbar boutons. As the CS axons also arise from neurons in layer V, such normalization procedure is likely to reflect the extent of layer V which was indeed invaded by BDA, thus taking into account the probable zone of tracer uptake (see Fregosi et al., 2017; Fregosi & Rouiller, 2017 for more detail). The pros and cons (limits) of the normalization procedure were discussed in detail in two recent reports (Fregosi & Rouiller, 2017; Fregosi et al., 2017), and will not be repeated here.

Statistical analysis was performed to compare the numbers of corticobulbar axonal boutons ipsilaterally versus contralaterally with respect to the injected hemisphere, when considering their total numbers (Figure 3) and within individual brainstem nuclei or group of nuclei (Supporting information Figure [Link], [Link]). These bilateral comparisons were based on parametric paired t‐test (and nonparametric Wilcoxon when required) applied to each of the consecutive individual sections analysed along the rostrocaudal axis (usually 12 sections overall, as in Fregosi et al., 2017; see also legend to Figure 3 and Supporting informationFigure [Link], [Link]).

## 3 RESULTS

**Figure 2:**
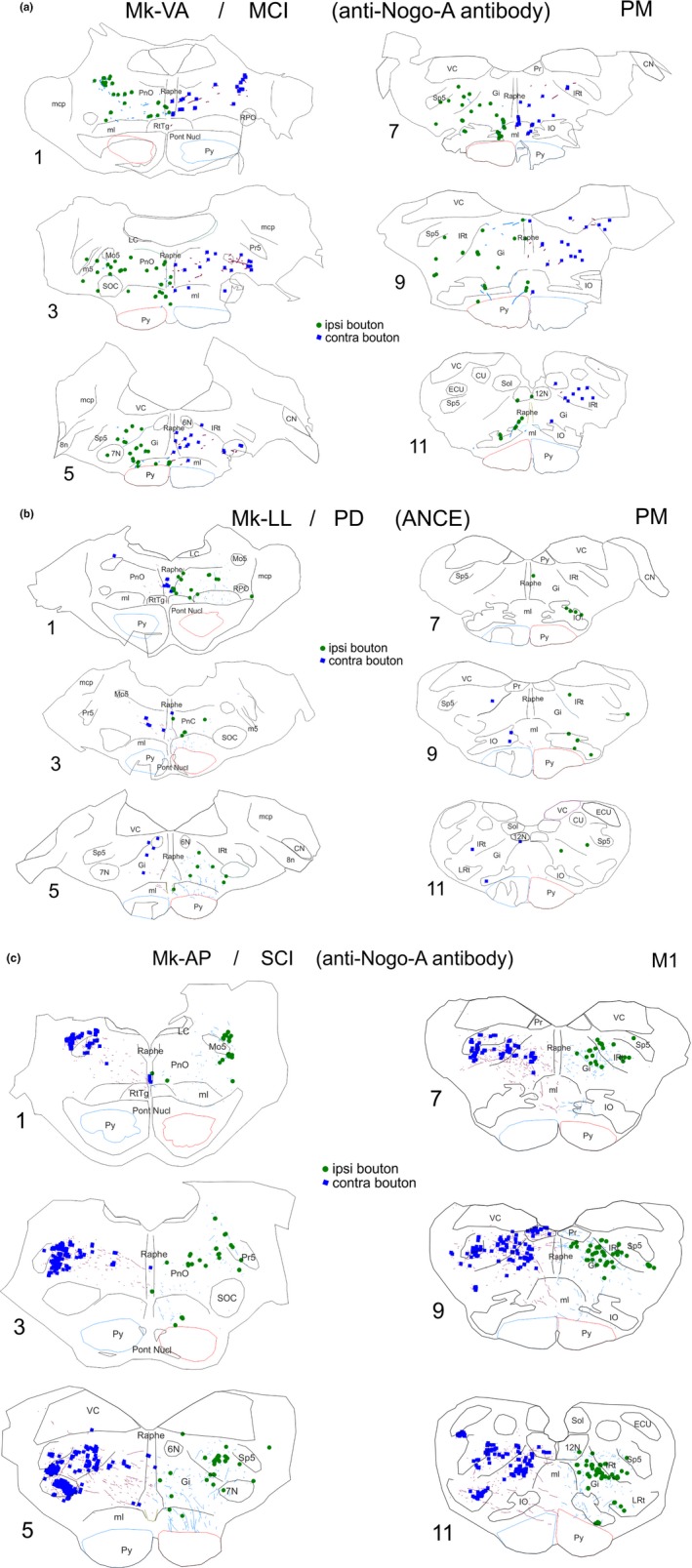
Typical distribution of corticobulbar axonal boutons in PMRF in six representative histological sections taken from an MCI monkey (panel a), from a PD monkey (panel b) and from an SCI monkey (panel c). Boutons in the ipsilateral PMRF are depicted in green, whereas those in the contralateral PMRF are depicted in blue. In addition, BDA‐labelled stem axons are shown in blue on the ipsilateral side and in purple contralaterally. For each monkey, the applied treatment is indicated in parentheses. On each section, the pyramid (Py), when outlined in red, corresponds to the ipsilateral side, with respect to the hemisphere injected with BDA (see Figure 1). See list of abbreviations

Thirteen animals were injected with the anterograde tracer BDA in either PM (*n* = 6) or M1 (*n* = 7), as listed in Table 1, in order to quantify the boutons emitted by the motor corticobulbar projections in PMRF. All animals subjected to cortical M1 lesion (*n* = 4) were injected in PM (in both PMd and PMv, except Mk‐RO with an injection in PMd only); all animals subjected to SCI (*n* = 5) were injected with BDA in M1, whereas, out of four animals subjected to parkinsonian lesion (*n* = 4), two were injected with BDA in PM (PMd and PMv) and two in M1. Figure 1 shows the reconstructions of the BDA injections sites in the 13 monkeys involved in the present study. Typical anterograde BDA labelling of corticobulbar axonal boutons in PMRF is shown in Figure 2 for three representative monkeys.

The 13 monkeys involved in the present anatomical analysis of the corticobulbar projections were also enrolled in behavioural assessment of their motor capacity, mainly manual dexterity for SCI and MCI monkeys, while a more global motor evaluation was conducted in MPTP monkeys. In order to contextualize the present anatomical data with behavioural performance, Table 2 provides a survey of functional recovery extent postlesion (SCI, MCI or MPTP) for each monkey.

**Table 2:** Percentages of functional recovery (“recov”) from the lesion (SCI, MCI or MPTP) assessed using various behavioural motor tasks

Monkeys ID	Spinal cord injured (SCI) monkeys				
	Mk‐CG	Mk‐CP	Mk‐AC	Mk‐AP	Mk‐AG
Treatment	Control Ab	Control AB	Anti‐Nogo‐A‐Ab	Anti‐Nogo‐A‐Ab	Anti‐Nogo‐A‐Ab
% recov vert	95	88	100	100	100
% recov horiz	85	83	100	95	100
% recov total	90	83	100	99	100

Monkeys ID	Motor cortex injured (MCI) monkeys			
	Mk‐MO	Mk‐VA	Mk‐RO	Mk‐BI
Treatment	Anti‐Nogo‐A‐Ab	Anti‐Nogo‐A‐Ab	Untreated	Untreated
% recov vert	84	87	100	94
% recov horiz	60	91	90	36
% recov total	76	87	98	74
% recov box	90	86	89	49

	MPTP monkeys (all ANCE treated)			
	Mk‐LY	Mk‐MI	Mk‐LL	Mk‐MY
% recov Sch‐s^b^	100	80	100	100
% impr fr‐a	21	9	18	9
% impr dist	16	70	38	45

Note

Manual dexterity was assessed in SCI and MCI monkeys based on the “modified Brinkman Board” task (e.g. Schmidlin et al., 2011) considering the scorea separately for the vertical slots (Vert), or the horizontal slots (Horiz) or grouping them (Total: all slots). In MCI monkeys, an additional task was considered, the Brinkman box (Hamadjida et al., 2012), in which the total time (in seconds) necessary to visit all slots (*n* = 20) was measured. In both SCI and MCI, the lesion resulted in total loss of manual dexterity (scores = 0; infinite total time) in the acute postlesion phase, lasting a few weeks. The percentage of recovery was assessed at the level of the postlesion plateau (with reference to prelesion plateau).

In MPTP monkeys, the percentage of functional recovery (“recov”) or improvement (“impr”) was assessed based on the Schneider score (Sch‐s), the time spent in freezing activity (fr‐a) and the traveled distance (dist). The percentage of improvement was measured by comparing the median values post‐ANCE implantation versus the post‐MPTP lesion (time window before ANCE; see Borgognon et al., 2017): less time in freezing activity, more distance travelled. Manual dexterity data in MPTP monkeys will be published later. Functional recovery data are taken from previous studies (Freund et al., 2009; Wannier‐Morino et al., 2008; Beaud et al., 2008, 2012; Wyss et al., 2013; Borgognon et al., 2017) . The percentage of recovery is to some extent related to the lesion volume (see lesion volume data in Table 1).

^a^Number of pellets successfully retrieved in 30 s. ^b^In the Schneider‐score, the percentage of recovery represented the proportion of the total deficit present after MPTP lesion which disappeared after autologous cells implantation. For instance, a Schneider score of 5 postlesion which decreased to 1 postimplantation corresponded to an 80% recovery.

### 3.1 Brief summary of the corticobulbar projection in intact monkeys

**Figure 3:**
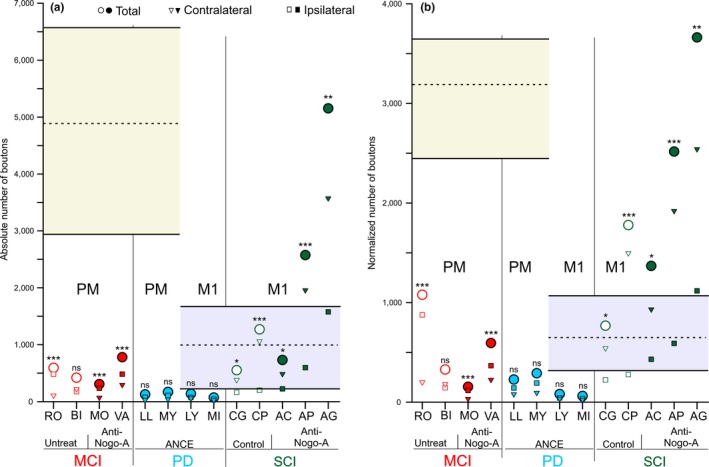
Scatter plots of the numbers of corticobulbar boutons observed in the different groups of monkeys subjected to motor cortex lesion (MCI), spinal cord injury (SCI) or MPTP intoxication (PD). For comparison, the data in intact monkeys (Fregosi et al., 2017) are represented by the range (yellow or light blue area) and the mean value (dashed horizontal line). The yellow area is for data in intact monkeys as a result of BDA injections in PM, whereas the blue area is for data in intact monkeys as a result of BDA injection in M1. In the monkeys subjected to MCI, SCI or PD, the BDA injection site (PM or M1) is indicated below the graph. Panel a is for the absolute numbers of corticobulbar boutons, whereas the panel b is for normalized numbers of corticobulbar boutons. Different symbols display either the total or individual numbers of boutons for the ipsilateral or contralateral PMRF (see legend on top of each panel). The presence/absence of treatment is indicated by filled or open symbols: filled symbols for anti‐Nogo‐A antibody in MCI or SCI monkeys as well as ANCE treatment in PD monkeys, and open symbols for untreated MCI monkeys or control antibody‐treated SCI monkeys respectively. Asterisks above the circle symbols indicate that the numbers of axonal boutons in PMRF were significantly different between the ipsilateral and contralateral sides of PMRF: *p <* *0.05, **p <* *0.01, ***p ≤ 0.001, ns is for nonstatistically significant difference between the two PMRF sides

The present study aims at assessing possible changes of the corticobulbar projection in adult monkeys following lesion, such as MCI, SCI or MPTP. This requires a comparison with the corticobulbar projection in intact monkeys, as reported recently (Fregosi et al., 2017). The normal corticobulbar projection to the PMRFis briefly summarized here. First, the corticobulbar projection was found to be clearly denser when originating from PM or the supplementary motor area (SMA) than from M1. This is clearly illustrated in Figure 3 with the comparison of corticobulbar boutons in PMRF originating from PM versus M1 (yellow vs. blue areas, respectively). Second, irrespective of the origin, the corticobulbar projection was bilateral although with a predominance on the ipsilateral PMRF when originating from PM or SMA and on the contralateral PMRF when coming from M1. Third, in PMRF, the main nuclei targeted by the corticobulbar projection were the pontine reticular nucleus pars caudalis and oralis (PnC and PnO), the gigantocellular reticular nucleus (Gi) as well as the lateral and intermediate reticular nuclei (LRt and IRt).

### 3.2 Corticobulbar projections from PM after motor cortical injury (MCI)

The quantitative data for the corticobulbar axonal boutons in PMRF originating from PM in the four MCI monkeys are available in Table 3. Independently of the treatment, after a lesion of M1 hand area, the corticobulbar projections arising from the ipsilesional PM strongly decreased in PMRF in all four MCI monkeys, as compared to intact monkeys (Figure 3a). The strong decrease in the density of corticobulbar projection from PM in MCI monkeys was confirmed based on the normalized number of boutons (Figure 3b). Figure 3a also indicates for each monkey the bilateral distribution of corticobulbar boutons in PMRF (ipsilateral vs. contralateral with respect to the BDA‐injected hemisphere), together with the related statistical analysis. Out of the four MCI monkeys, three exhibited a statistically significant ipsilateral predominance (see also Table 3), as observed in two out of three intact monkeys (Fregosi et al., 2017).

**Table 3:** Corticobulbar projections from PM in MCI monkeys

	Mk‐MO^a^	Mk‐VA^a^	Mk‐RO	Mk‐BI
Total nb. of boutons	295	772	582	427
Ipsilateral boutons	225	475	473	185
Contralateral boutons	70	297	109	242
% Ipsilateral	**76.3%**	**61.5%**	**81.3%**	**43.3%**
% Contralateral	**23.7%**	**38.5%**	**18.7%**	**56.7%**
Normalized nb. of boutons*1,000 Ipsi	114	362	871	139
Normalized nb. of boutons*1,000 Contra	35	226	201	182

Note

Numbers (nb.) of corticobulbar boutons (*en passant* and *terminaux*) in the brainstem given in absolute values (top three rows) and in normalized values (bottom two rows) for animals with cortical lesion in M1 hand area (MCI). The global percentages of boutons on the ipsilateral and contralateral sides are given in Bold. In all four monkeys, BDA was injected in PM. The monkeys Mk‐MO and Mk‐VA received the anti‐Nogo‐A antibody treatment^a^, whereas the monkeys Mk‐RO and Mk‐BI were untreated. The numbers of labelled CS axons used for normalization in each monkey are given in Table 1.

As far as the target nuclei in PMRF are concerned (Supporting information Figure S1a), the four MCI monkeys exhibited some individual variability (as previously observed in intact monkeys), although it remained that the pontine reticular nuclei (PnO+PnC), the gigantocellular reticular nucleus (Gi) and the lateral/intermediate reticular nuclei (LRt+IRt) are the main target nuclei in PMRF. There was, however, a tendency towards a slight increase of the corticobulbar projection to the raphe nuclei in the MCI monkeys (Supporting information Figure S1a) as compared to intact monkeys (Fregosi et al., 2017). Looking at the rostro‐caudal distribution of the corticobulbar boutons in PMRF for the MCI monkeys (Supporting information Figure S2a), they were generally more abundant in the rostral half of PMRF; this rostro‐caudal distribution also confirms the general ipsilateral dominance, mentioned above based on Figure 3a and Table 3.

Considering the volume of the lesion in M1, there was a slight trend towards a greater decrease in the density of the corticobulbar projection to PMRF originating from PM for increasing lesion size in M1 (Supporting information Figure S3a). However, this observation is limited by the small number of monkeys subjected to MCI (*n* = 4). Regarding the treatment (anti‐Nogo‐A antibody), the two treated monkeys exhibited a comparable decrease of the corticobulbar projection from PM as the two untreated monkeys (Figure 3; Supporting information Figure S3a), taking the intact monkeys as reference. In other words, in case of MCI, the anti‐Nogo‐A antibody treatment did not impact on the decreasing effect of the M1 lesion on the corticobulbar projection from PM, although again, the number of cases is limited.

### 3.3 Corticobulbar projections from PM and M1 after MPTP lesion (PD monkeys)

The PD monkeys Mk‐LL and Mk‐MY received BDA injections in both PMd and PMv, whereas Mk‐LY and Mk‐MI were injected with BDA in M1 (Figure 1). The detailed quantitative data are available in Table 4 for these four monkeys. A dramatic effect in the PD monkeys was observed for the corticobulbar projection from PM, which was substantially decreased as compared to intact monkeys (Figure 3a). A decrease was also observed in PD monkeys for the corticobulbar projection originating from M1, but apparently to a lesser extent than from PM (Figure 3a), although proportionally to the density in intact monkeys the decrease of the projection from M1 appears also strong (Figure 3a). The same changes in corticobulbar projections from PM and M1 in PD monkeys were maintained in the normalized data (Figure 3b).

**Table 4:** Corticobulbar projections from PM or M1 in PD monkeys

	PM injections		M1 injections	
	Mk‐LL	Mk‐MY	Mk‐LY	Mk‐MI
Total nb. of boutons	132	174	127	62
Ipsilateral boutons	83	115	66	34
Contralateral boutons	49	59	61	28
% Ipsilateral	**62.9%**	**66.1%**	**52.0%**	**54.8%**
% Contralateral	**37.1%**	**33.9%**	**48.0%**	**45.2%**
Normalized nb. of boutons*1,000 Ipsi	140	188	39	30
Normalized nb. of boutons*1,000 Contra	83	97	37	25

Note

Numbers (nb.) of boutons (*en passant* and *terminaux*) in the brainstem given in absolute values (top three rows) and in normalized values (bottom two rows) in PD animals. The global percentages of boutons on the ipsilateral and contralateral sides are given in bold. BDA was injected in PM in two monkeys (Mk‐LL, Mk‐MY), whereas it was injected in M1 in the other two monkeys (Mk‐LY, Mk‐MI). All monkeys were subjected to the ANCE treatment. The numbers of labelled CS axons used for normalization in each monkey are given in Table 1.

The corticobulbar projections to PMRF from PM in MPTP monkeys showed no statistically significant bilateral difference towards any side of the brainstem; the same is true for the projections from M1 (Figure 3a). There were globally more boutons on the ipsilateral side in the four PD monkeys (Table 4), but the differences were not statistically significant based on paired sections’ comparison (Figure 3b). As compared to intact monkeys, bilateral predominance observed in intact monkeys was thus eliminated after MPTP lesion.

The distribution of the boutons across the nuclei of PMRF (Supporting information Figure S1b) revealed that they were located in the same three main targets nuclei (Pn, Gi, Rt), as in the intact monkeys and MCI monkeys. As the latter group, there was also a tendency to observe more boutons in the raphe nuclei in PD monkeys than in intact animals (Fregosi et al., 2017). Moreover, as compared to MCI monkeys, in PD monkeys the boutons were somewhat more homogeneously distributed along the rostro‐caudal axis of PMRF after injection in M1 (Supporting information Figure S2b).

The normalized amount of boutons in PMRF in the four PD monkeys was confronted to the percentage loss of dopaminergic neurons in the SNpc (Table 1; Supporting information Figure S3B). One animal (Mk‐LY) exhibited a loss of 39%, whereas the other three PD monkeys had a loss of 67%–74%. Nevertheless, all four PD monkeys exhibited a comparable number of corticobulbar boutons in PMRF, suggesting that at about 40% loss or more, the decrease of the density of the corticobulbar projection is large and comparable (floor level).

### 3.4 Corticobulbar projections from M1 after spinal cord injury (SCI)

Five monkeys subjected to SCI, namely, lateral cervical cord hemi‐section (Mk‐CG, Mk‐CP, Mk‐AC, Mk‐AP and Mk‐AG) were injected with BDA in M1 (Figure 1). The two monkeys Mk‐CG and Mk‐CP were treated for 4 weeks intrathecally with a control antibody, whereas the other three monkeys were infused with the anti‐Nogo‐A antibody (Table 1). The quantitative data on the corticobulbar projection to PMRF from M1 in these five monkeys are available in Table 5. The SCI lesion had a different impact on the corticobulbar projection than MCI or PD. Indeed, there was no decrease of the density of the corticobulbar projection from M1 but rather a maintenance or a moderate increase in the control antibody treated monkeys, whereas following anti‐Nogo‐A antibody treatment, the density of the corticobulbar projection from M1 was substantially increased in two out of three monkeys (Figure 3a, b). In all five SCI monkeys, there was a significant bilateral bias towards the contralateral PMRF (Figure 3a; Table 5), as was the case in intact monkeys after BDA injection in M1 (Fregosi et al., 2017).

**Table 5:** Corticobulbar projections from M1 in SCI monkeys

	Mk‐CG	Mk‐CP	Mk‐AC^a^	Mk‐AP^a^	Mk‐AG^a^
Total nb. of boutons	546	1,263	733	2,576	5,142
Ipsilateral boutons	157	195	230	603	1,566
Contralateral boutons	389	1,068	503	1,973	3,576
% Ipsilateral	**28.8%**	**15.4%**	**31.4%**	**23.4%**	**30.5**
% Contralateral	**71.2%**	**84.6%**	**68.6%**	**76.6%**	**69.5**
Normalized nb. of boutons*1,000 Ipsi	219	274	428	589	1,115
Normalized nb. of boutons*1,000 Contra	543	1,500	937	1,927	2,545

Note

Numbers (nb.) of boutons (*en passant* and *terminaux*) along the brainstem given in absolute value (three top rows) and in normalized values (two bottom rows) in SCI animals. The global percentages of boutons in the ipsilateral and contralateral sides are given in bold. BDA was injected in M1 in all five monkeys. The monkeys Mk‐CG and Mk‐CP received a control antibody, whereas the other three monkeys^a^ were treated with the anti‐Nogo‐A antibody. The numbers of labelled CS axons used for normalization in each monkey are given in Table 1.

With respect to the nuclear distribution of boutons in PMRF, they were numerous in the nuclei Gi and Rt in the five SCI monkeys, as in intact monkeys, but less in the nucleus Pn than in intact monkeys (Supporting information Figure S1c). In contrast to the MCI and PD groups, there was no trend for an increase of the number of boutons in the raphe nuclei in the five SCI monkeys (Supporting information Figure S1c). Along the rostro‐caudal axis of PMRF (Supporting information Figure S2c), there was in the five SCI monkeys a large interindividual variability, although the distribution confirms the contralateral predominance. Finally, the number of boutons in the SCI monkeys did not show a systematic relation with the extent of the cervical lesion (Supporting information Figure S3c).

## 4 DISCUSSION

### 4.1 Summary of the main findings

The present data first demonstrate that after cortical lesion of M1 hand area the density of corticobulbar projections from PM onto the PMRF strongly decreased (Supporting information Figure S3 **)**, but nevertheless largely maintained their ipsilateral predominance present in intact animals (Table 3). Second, the corticobulbar projections from PM in animals affected by MPTP lesion mimicking PD (and treated with ANCE) showed a strong decrease in density (Figure 3) with a maintenance of ipsilateral predominance (Table 4); similarly, also in PD monkeys there was a decrease of the density of the corticobulbar projections from M1 (Figure 3), but to a lesser extent than from PM, and associated with a loss of contralateral lateralization (Table 4). Third, monkeys subjected to cervical cord hemi‐section at C7 level exhibited an increase of the corticobulbar projections from M1 onto the PMRF after a treatment with anti‐Nogo‐A antibody (Figure 3) with a maintenance of a contralateral predominance (Table 5); in contrast, SCI monkeys treated with a control antibody did not show such an increase of corticobulbar projection density from M1, whereas the contralateral predominance was preserved.

### 4.2 Limitations of the present study

A first limitation of the present study is obviously the fairly low numbers of monkeys included in each group of pathologies, although this is common in nonhuman primate studies for ethical reasons. Also, in order to minimize the number of monkeys involved, this study capitalized on previously available cases, initially designed to test various therapies applied to MCI, PD or SCI. Furthermore, in each group of pathologies, the animals were further subdivided into treated versus control (or untreated) animals, at least in the motor cortex lesion and SCI groups. Such small numbers in groups and subgroups prevent reliable and robust correlations of changes in the number of corticobulbar axonal boutons with lesion size (SCI or motor cortex lesion) or neuronal loss (dopaminergic cells in SNpc in PD monkeys) and consequently any indirect inference as to whether the lesion‐related adaptation of the corticobulbar projection may have played a role in the functional recovery. For instance, following M1 lesion, the trend towards less corticobulbar boutons for increasing lesion volumes (Supporting information Figure S3a) has to be taken with great caution.

A further limitation is the variability in the motor cortical area injected with the tracer BDA. The choice to inject BDA in PM in monkeys subjected to M1 lesion was dictated by the evidence that PM plays an important role in the functional recovery from M1 lesion (Hoogewoud et al., 2013; Liu & Rouiller, 1999). In SCI monkeys, M1 only was injected with BDA as the initial goal in these animals was to study the reorganization of the CS projection originating mainly from M1 (Freund et al., 2007). In other words, the present study does not address the possibility of a change in CS projection originating from PM in SCI monkeys. In PD monkeys, although BDA was injected in PM or in M1, there are only two monkeys in each subgroup of BDA injections.

The corticobulbar projections in intact monkeys was shown to be denser when originating from PM and SMA than when originating from M1 (Fregosi et al., 2017). As a result, in the present study, a possible decrease of the density of the corticobulbar projections due to a lesion could be better detected when originating from PM than from M1. Moreover, this study lacks cases injected with BDA in SMA. Lesion‐related changes affecting the corticobulbar projections from SMA are nevertheless likely, considering the quantitative importance of supraspinal projections originating from SMA (Fregosi et al., 2017; Macpherson, Wiesendanger, Marangoz, & Miles, 1982) and the changes of the CS projection from SMA reported after a cortical lesion involving M1 and the lateral PM (McNeal et al., 2010; Morecraft et al., 2015). Another limitation of the present study in the group of PD monkeys (*n* = 4) is that they were all subjected to the ANCE treatment, thus missing a subgroup of MPTP‐lesioned monkeys without treatment. As a consequence, the decrease of the corticobulbar projection observed in the PD monkeys (especially that originating from PM) may result from the MPTP lesion, the ANCE treatment, or both.

The plastic changes observed for the corticobulbar projections onto the PMRF as a result of M1 lesion or SCI or PD reflect the state of the connectivity taken at a single time point, which is several months (range 3–8 months) postlesion or MPTP intoxication when the monkeys reached a plateau of usually incomplete functional recovery (see Kaeser et al., 2011; Wyss et al., 2013; Freund et al., 2006, 2009; Borgognon et al., 2017 for behavioural data). The pattern of corticobulbar connectivity may not be the same at an earlier time point, especially during the acute phase of functional recovery before reaching the plateau. We therefore cannot exclude that the corticobulbar projection pattern (density and laterality) dynamically varies during the consecutive phases of the functional recovery, for instance in the form of a sequence of sprouting followed by pruning (see e.g., Pernet & Schwab, 2012 for review).

The comparison of the corticobulbar projection from PM versus M1 is based on a given volume of cortical tissue injected with BDA. However, the total volume of the M1 cortical area is larger than the PM cortical area, although it is difficult to estimate how much larger, as the borders between the motor cortical areas are not strict, but rather correspond to progressive functional transition zones. In other words, if considering the entire M1, would the total number of corticobulbar projections from M1 be closer to that from PM? To take into account this difference in total cortical area volume, in the intact monkeys (Fregosi et al., 2017) the BDA injections in M1 in two out of three monkeys were about three times larger than those in PM (still slightly larger in the third animal). In the present study, in both groups MCI and SCI (Table 1), the volumes of BDA injected in PM and M1, respectively, were comparable to those injected in PM and M1 in intact animals (for M1 comparable to the two large injections in intact monkeys). Larger BDA injections in M1 than in PM then contribute to attenuate, at least to some extent, the difference in total volume between these two motor cortical areas, reducing the risk of a bias towards PM. In the present study, the exceptions are the MPTP monkeys (PD group) in which the volume of BDA injected in M1 was lower, comparable to that injected in PM (Table 1). One cannot exclude the possibility that the moderate decrease of corticobulbar projection from M1 in PD monkeys as compared to intact monkeys (Figure 3) is due to smaller BDA injection sites.

### 4.3 Changes of corticobulbar projections as a function of lesion type and treatment

The plastic changes of the corticobulbar projections from PM or M1 resulting from a CNS lesion are different for each type of pathology (MCI, PD, SCI). They may be interpreted in the context of a wide but tightly interrelated system of multiple descending projections, all cooperating to the control of unilateral voluntary movements and exhibiting various laterality properties. It includes the corticospinal, corticothalamic, corticorubral, corticobulbar (corticoreticular), corticotectal, rubrospinal, reticulospinal, vestibulospinal, tectospinal projections. On top of these, there are the motor corticocortical connections (intra‐ and interhemispheric), the motor loops via the basal ganglia and cerebellum, all contributing also importantly to the shaping of voluntary movements. In case of lesion, the subtle function of this complex system of multiple projections and loops is challenged, requesting an adaptation in order to reestablish the best possible motor control. To this aim, one can speculate that spontaneous plasticity (in absence of treatment) may lead to a rearrangement within this system, by reducing some projections and increasing others, though within a restricted range as the adult CNS has limited plastic capacity, especially in terms of axonal regrowth (Schwab, 2010; Schwab & Strittmatter, 2014). The present data show that, following brain lesion (MCI or PD), there is a general adaptation of the system towards a decrease of the remote corticobulbar projections, originating from PM in case of MCI and coming from both PM and M1 in case of PD. Whether this distant corticobulbar adaptation contributes to the (incomplete) functional recovery remains an open question, as well as to what extent all the other subsystems of projections are also modified in parallel. In case of application of treatments, one may expect different types of postlesion adaptation of the overall motor system, either in the sense of emphasizing the spontaneous changes or implementing a different balance between the multiple projections subsystems. After unilateral M1 lesion, the callosal projections reaching the ipsilesional PM were enhanced by the anti‐Nogo‐A antibody treatment, especially the homotopic one coming from the opposite PM (Hamadjida et al., 2012). In the present study, following unilateral M1 lesion, the corticobulbar projection from the ipsilesional PM spontaneously decreased, an adaptation which was not impacted in one direction or the other by the anti‐Nogo‐A antibody treatment (Figure 3). In the case of PD, there was a strong decrease of the corticobulbar projection from both PM and M1 (Figure 3). A signal needs to be sent to the motor cortex in order to generate an adaptation (decrease) of the corticobulbar projection density in the PD monkeys. By reducing the number of dopaminergic neurons subcortically (mainly in SNpc, but not only), the MPTP lesion is also likely to decrease the diffuse dopamine innervation of the cerebral cortex at large (Gaspar, Duyckaerts, Alvarez, Javoy‐Agid, & Berger, 1991; Rosenberg & Lewis, 1995; Sesack, Snyder, & Lewis, 1995), including the motor cortex. This may represent the plasticity signal through which the PD lesion may trigger the decrease of the corticobulbar projection density, as compared to intact monkeys. The ANCE treatment may also play a role in such plasticity process, as the reimplanted ANCE cells produce growth factors (Bloch et al., 2014; Brunet et al., 2009) where they have been injected (in the striatum), but which can diffuse up to cerebral cortex.

As far as the animals subjected to SCI are concerned, the cervical cord hemi‐section did not generate any spontaneous change of the corticobulbar projection. However, the present study provides evidence that the anti‐Nogo‐A antibody treatment can significantly influence, as the corticobulbar projection from M1 was enhanced in the anti‐Nogo‐A antibody treated monkeys as compared to control antibody‐treated monkeys (Figure 3a). With that respect, the corticobulbar projection from M1 behaves similarly to the CS projection following SCI (Freund et al., 2007).

How to interpret the opposite effects of the same anti‐Nogo‐A antibody treatment on the corticobulbar projection following either lesion of M1 or SCI (Figure 3a)? It can be hypothesized that the axonal collateralization plays an important role. About one‐third of the corticobulbar projection consists of collaterals emitted by CS axons on their way to the spinal cord (Keizer & Kuypers, 1989). The corresponding neurons of origin in layer V were therefore impacted by the SCI, which may have represented a retrograde “damage signal”, triggering the anti‐Nogo‐A antibody to promote sprouting in PMRF in this specific subpopulation of M1 neurons. In contrast, in the case of MCI, the neurons of PM projecting to PMRF may not have been impacted directly by the cortical lesion, possibly explaining the absence of a trigger initiating the axonal sprouting in PMRF. On the level of molecular mechanisms, there might also be differences between M1 and PM neurons and axons with regard to Nogo‐A receptors and responses to the neutralization of endogenous Nogo‐A.

The corticobulbar projection terminating in the PMRF is functionally acting as a cortical regulator of the reticulospinal tract (RST) projection system. As a consequence, when the density of the corticobulbar projection is decreased as a result of M1 lesion or MPTP lesion, the regulation from the cortex on the RST system is also reduced, leading to a more autonomous influence of the RST system on the spinal cord and motor output, which may be part of the functional recovery mechanisms. In other words, if the corticobulbar projection normally exerts an inhibitory effect, then a decrease in its density after MCI or MPTP lesion would lead to disinhibition of the cells of origin of the reticulospinal system. Such adaptation would correspond to a release mechanism, assisting reticulospinal‐mediated movements. On the contrary, in case of SCI and in the absence of treatment (control antibody cases) the cortical regulation of the RST system is largely unchanged (Mk‐CG and Mk‐CP in Figure 3). However, when the SCI monkeys received the anti‐Nogo‐A antibody treatment, the corticobulbar projection was enhanced (Figure 3), in at least two out of three monkeys, indicating that the motor cortex (M1) has increased its regulation on the RST system, potentially via its indirect influence on the spinal cord and motor output. This increased indirect influence may substitute part of the lost CS projection, representing a mechanism of functional recovery. The present increase of corticobulbar projection after SCI and anti‐Nogo‐A antibody treatment is reminiscent of an increase in density of other corticofugal projections observed in rodents, onto the red nucleus and pontine nuclei, after unilateral pyramidotomy or stroke and administration of anti‐Nogo‐A antibodies (Bachmann, Lindau, Felder, & Schwab, 2014; Seymour et al., 2005; Z'Graggen, Metz, Kartje, Thallmair, & Schwab, 1998). In the three monkeys subjected to SCI and anti‐Nogo‐A antibody treatment, two of them exhibited a strong increase of the corticobulbar projection density from M1, which was much less in the third monkey Mk‐AC (Figure 3a). For the latter monkey, this result may be explained, at least in part, by the small size of the BDA injection in M1 and less well targeted to the hand area (Figure 1c).

### 4.4 Laterality of the corticobulbar projection

The complexity of the postlesion adaptive response of the overall system of multiple motor projections and loops is even amplified when considering their different laterality properties. The CS tract is clearly a predominantly crossed subsystem of projections (Lacroix et al., 2004; Rosenzweig et al., 2009), whereas the corticobulbar projection is more bilateral (Fregosi et al., 2017). The reticulospinal subsystem is also largely bilateral (Fregosi et al., 2017; Sakai et al., 2009).

In PD monkeys (symmetrical lesion) the two animals with BDA injections in PM exhibited a preservation of the ipsilateral predominance comparable to intact animals, whereas in contrast, the laterality in the two PD monkeys with BDA injections in M1 was modified in the form of a loss of contralateral predominance present in intact animals: 45%–48% contralateral corticobulbar projections in the two PD monkeys and 58%–71% in intact monkeys (Fregosi et al., 2017). On the other hand, after unilateral cervical hemi‐section and BDA injection in M1, the contralateral predominance of the corticobulbar projection from M1 in intact animals (58%–71%) was preserved if not slightly enhanced (68%–84%). Finally, following unilateral M1 lesion, the corticobulbar projection originating from PM remained predominantly ipsilateral, at least in three out of four monkeys, as in intact animals (Fregosi et al., 2017).

### 4.5 Corticobulbar projections onto lateral reticular (Rt), raphe nuclei and PMRF nuclei

The lateral tegmentum of the brainstem contains, together with the sensory nuclei, the lateral reticular formation (Paxinos et al., 2000), in which we identified the lateral reticular nucleus (LRt) and the intermediate reticular nucleus (IRt), together referred to as Rt nucleus (see also Alstermark & Ekerot, 2013 for a review). As compared to intact animals (Fregosi et al., 2017) and control SCI antibody‐treated monkeys, the SCI monkeys treated with anti‐Nogo‐A antibody exhibited increased projections from M1 to Rt nuclei in the monkeys Mk‐AP and Mk‐AG. This may strengthen indirect projections to the cerebellum and medial reticular formation, thus contributing to postlesion compensatory mechanisms.

In intact animals, the raphe nuclei receive bilateral projections from motor areas (Fregosi et al., 2017). The corticobulbar projections from motor areas to the raphe nuclei are also present after motor cortex or spinal cord lesions and PD, with projections from PM remaining slightly stronger than those from M1.

Besides expected individual variability, there was no systematic and major change of distribution when comparing the location of corticobulbar boutons in PMRF in the three groups of monkeys subjected to M1 lesion, or SCI or MPTP lesion (Supporting information Figure [Link], [Link]) with that of corticobulbar boutons in intact monkeys (Fregosi et al., 2017; : their figure 6). The exception is a substantial increase in the percentage of the corticobulbar boutons in the pontine reticular nuclei (PnO + PnC; Supporting information Figure [Link], [Link]) observed in the MPTP lesioned monkeys after BDA injection in M1.

### 4.6 Functional significance

There is still incomplete understanding of the precise role played by brainstem‐descending pathways in motor recovery from lesion, as well as by corticobulbar pathways. Although previous reports speculated that corticobulbar projections may play an important role in motor recovery after lesion of the CNS (Beaud et al., 2008; Hoogewoud et al., 2013; Mosberger et al., 2017; Wyss et al., 2013; Zörner & Schwab, 2010), this descending tract still remains mysterious for the most part, possibly due in part to its location and the difficult access to the corresponding target nuclei in the brainstem. The present study is original as it represents a unique study in nonhuman primates quantitatively investigating the rearrangement of the corticobulbar projections onto the PMRF following different pathologies affecting the CNS, such as motor cortex lesion, PD or SCI. The corticobulbar projections act as cortical modulator onto descending brainstem pathways, mainly the RST as far as the PMRF is concerned.

There is evidence in both nonhuman primates and humans that the RST is also involved in the control of distal muscles allowing crude hand motor function (Baker, 2011; Honeycutt et al., 2013; Riddle & Baker, 2010; Riddle et al., 2009; Soteropoulos et al., 2012). However, few investigations have assessed the role of the RST in nonhuman primates in motor recovery after stroke or medullary pyramidal lesion (Herbert et al., 2015; Zaaimi et al., 2012) and still none after spinal cord injury. After unilateral medullary CST lesion, flaccid paralysis of the hand was first observed that was then followed by some spontaneous functional recovery in which the RST was involved (Zaaimi et al., 2012). After cortical injury of shoulder/elbow M1 representation in monkeys, the RST was shown to play a role in the recovery of reaching movements (Herbert et al., 2015). Corresponding observations in rats were reported on the role of the RST in functional recovery in rodents after either cortical lesion (Bachmann et al., 2014) or spinal cord lesion (Ballermann & Fouad, 2006; Filli et al., 2014; Garcia‐Alias, Truong, Shah, Roy, & Edgerton, 2015; Zörner et al., 2014). In a rat model of spinal cord contusion, it was demonstrated that a reorganization of the cortico‐reticulo‐spinal circuit enabled functional recovery of walking and swimming (Asboth et al., 2018). Studies on mice showed that after stroke of the sensorimotor cortex there was an increase of the CST and RST projections as well as corticoreticular projections, with projections from RST six times stronger than those from CST (Bachmann et al., 2014). They observed an increased innervation of the nucleus Gi and Raphe nuclei from the contralesional cortex and, in turn, these nuclei were the main source of increased projections to the denervated spinal cord.

The regulation exerted by the motor cortical areas via their corticobulbar projections to PMRF on the RST system varies according first to the cortical area of origin in intact monkeys (denser from PM and SMA than M1: Fregosi et al., 2017) and, second, to the type of lesion/pathology as well as treatment administered (present study). The precise impact of the corticobulbar terminals, when activated, onto the RST neurons remains unknown: is it excitatory, inhibitory or both? The latter is most likely and in that case it would be crucial to know whether plastic changes induced by lesion/pathology and/or treatment are restricted to either excitatory or inhibitory synapses, or whether it is unspecific. This information is needed in order to interpret how the regulation of the motor cortical areas onto the RST is modified after lesion and administration of a treatment, an issue to be addressed in future studies. The motor cortex, the reticular formation and the spinal cord contribute altogether to the control of coordinated voluntary movements, including reaching and grasping (Zaaimi, Dean, & Baker, 2018). These authors demonstrated that the brainstem output is more efficient, but less precise in producing motor output than the motor cortex or the spinal cord, the latter two contributing to the refinement of gross muscles synergies produced by the reticular formation. The changes of the corticoreticular projections observed in this study following CNS lesions may thus represent an adaptation of the cortical influence on the intact reticular formation.

## CONFLICT OF INTEREST

The authors declare to have no conflict of interest in relation to this study, except that the anti‐Nogo‐A antibody was provided by Novartis AG.

## DATA ACCESSIBILITY

The histological material (sections) is publicly available at the SPCCR on request. The same applies to video sequences of the behaviour of the animals included in this study. The detailed reconstruction (equivalent of Figure 2) of the corticobulbar projection in each monkey is available in the Ph.D thesis manuscript of Michela Fregosi, available in the repository of Ph.D. theses completed at the Faculty of Sciences of University of Fribourg (http://opac.rero.ch/gateway?).

## AUTHOR'S CONTRIBUTIONS

EMR and MF designed the tracing analysis. MF, AC and EMR analysed the histological sections. SBa, SBo, JC, JFB, JB and EMR designed and performed the MPTP experiments. EMR and MES designed the anti‐Nogo‐A antibody treatments. MF and EMR drafted the manuscript.
