# Supplementary figures and images for: Changes of motor corticobulbar projections following different lesion types affecting the central nervous system in adult macaque monkeys
Source: Eur J Neurosci. 2018 Aug 16;48(4):2050–70. doi: 10.1111/ejn.14074 (PMC6175012; doi:10.1111/ejn.14074)

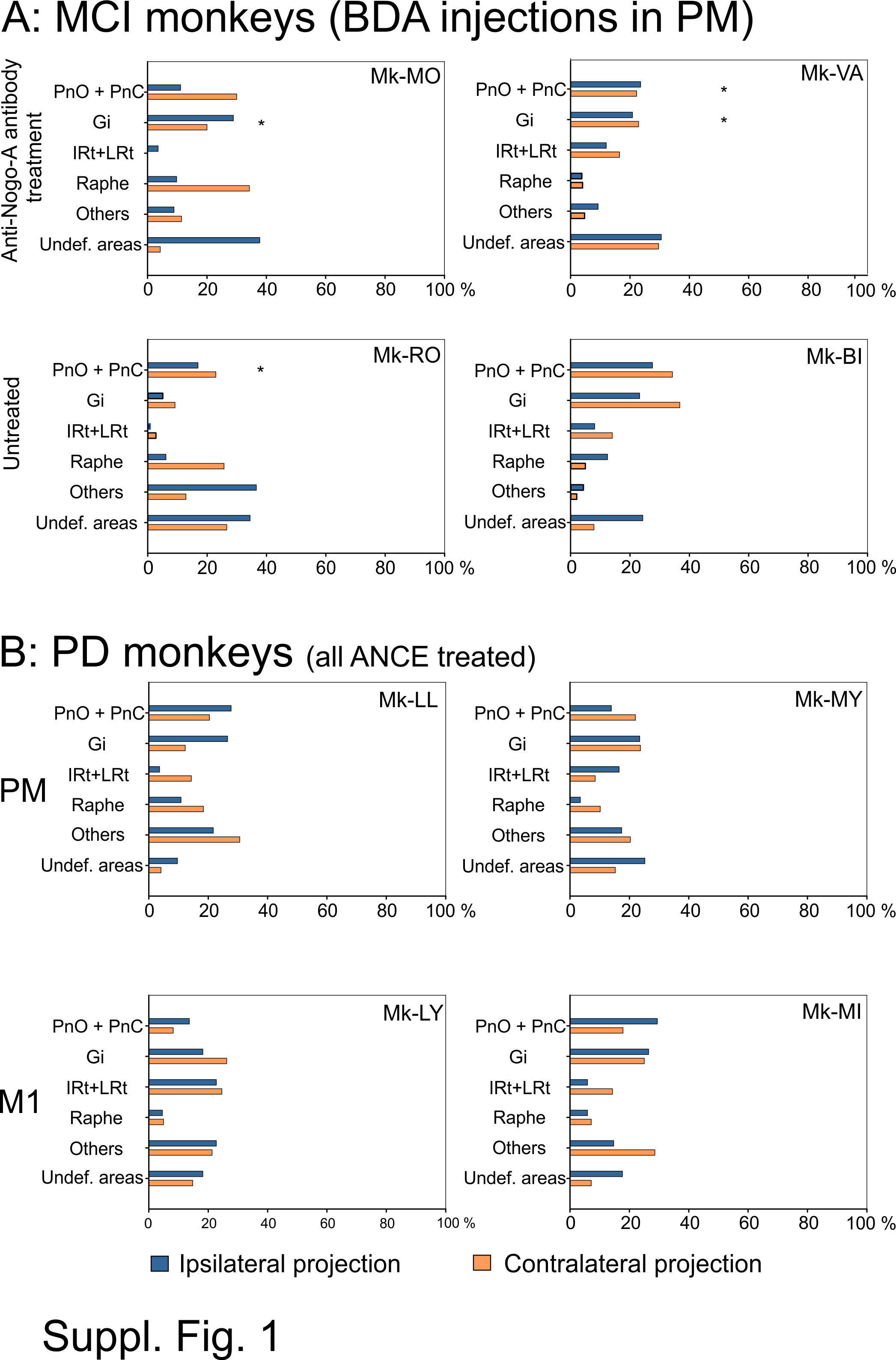

Supplement: Supplementary file 2 [file EJN-48-2050-s002.tif]

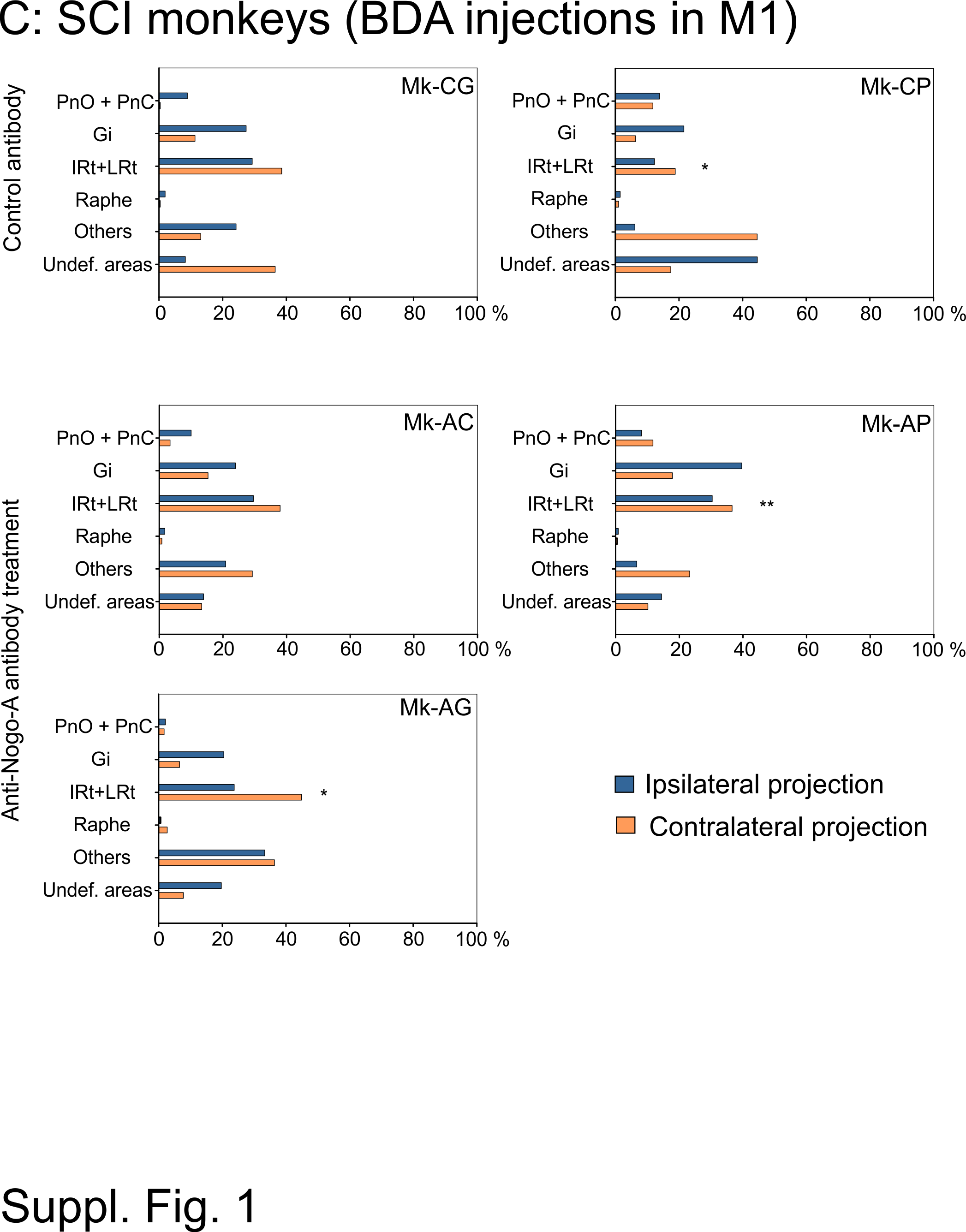

Supplement: Supplementary file 3 [file EJN-48-2050-s003.tif]

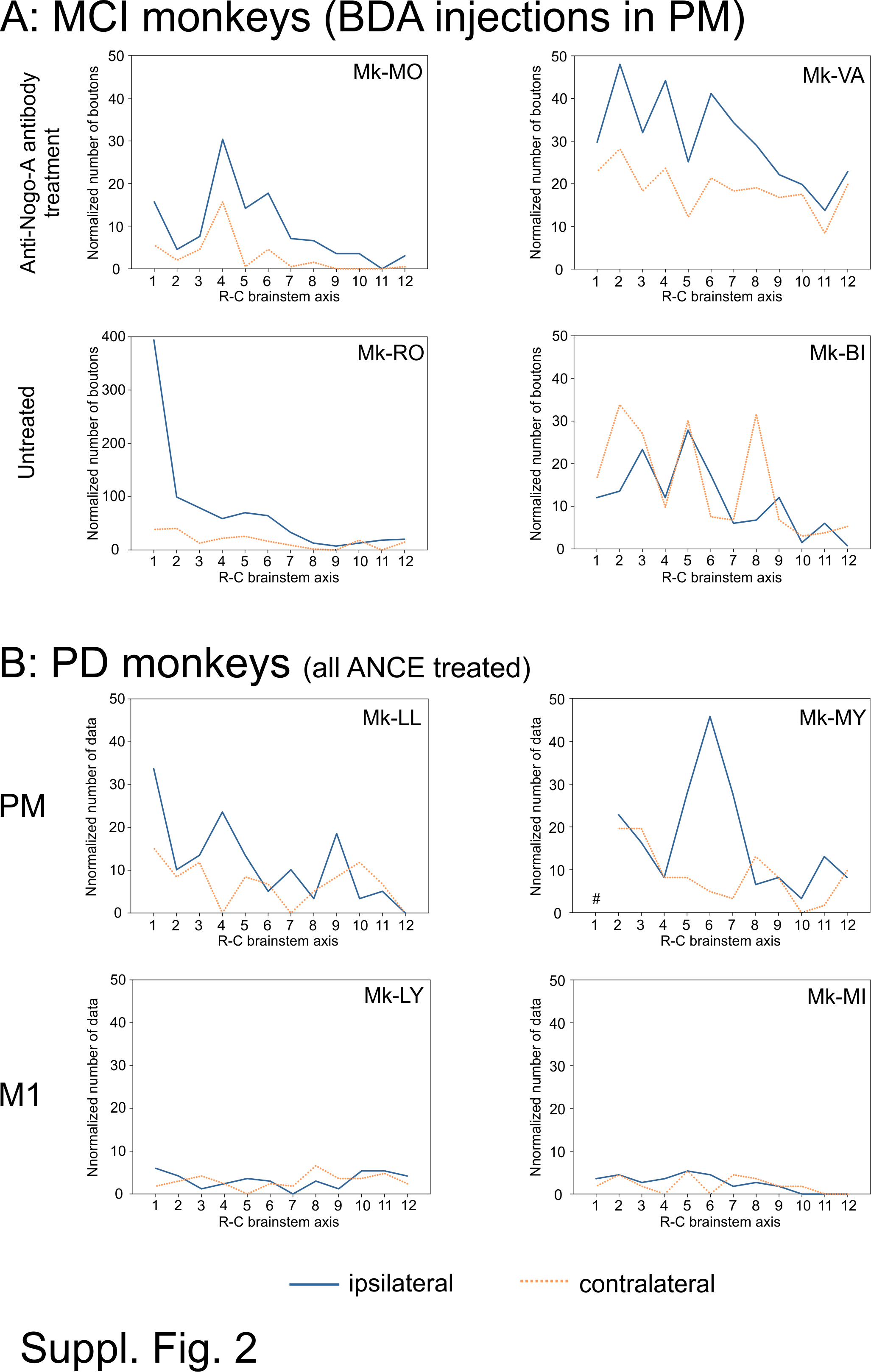

Supplement: Supplementary file 4 [file EJN-48-2050-s004.tif]

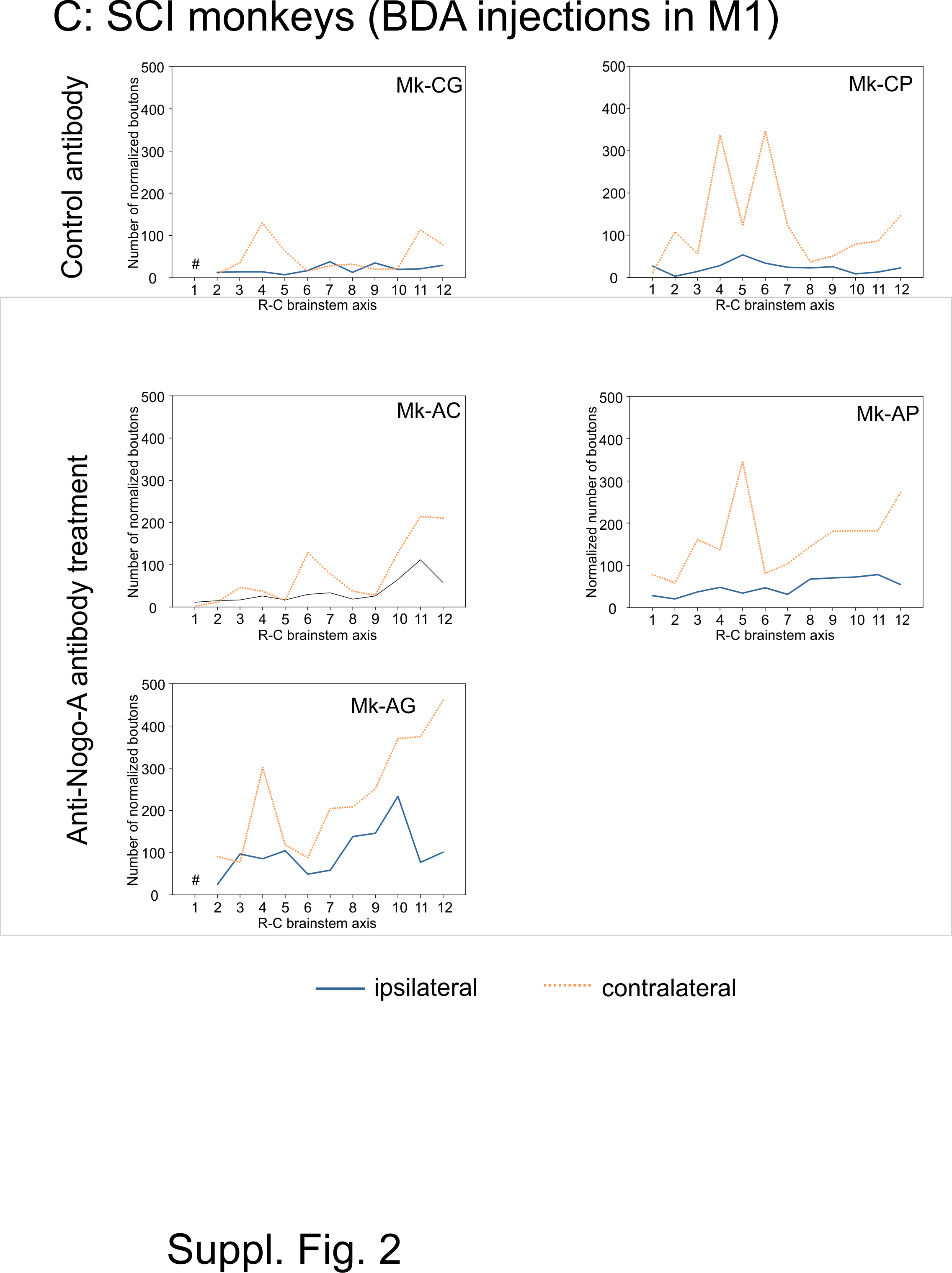

Supplement: Supplementary file 5 [file EJN-48-2050-s005.tif]

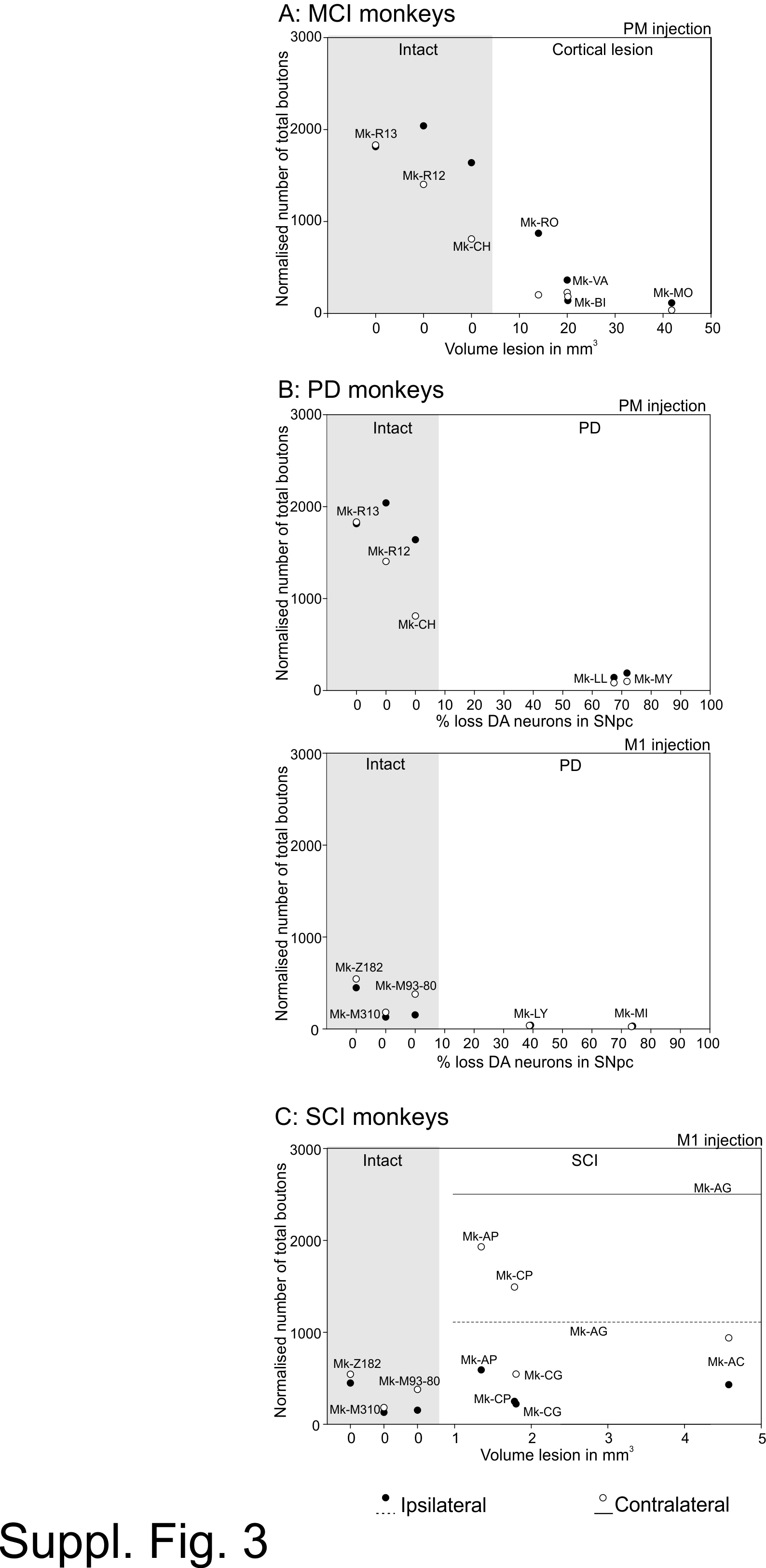

Supplement: Supplementary file 6 [file EJN-48-2050-s006.tif]
