# Supplementary material for: Changes of motor corticobulbar projections following different lesion types affecting the central nervous system in adult macaque monkeys
Source: Eur J Neurosci. 2018 Aug 16;48(4):2050–70. doi: 10.1111/ejn.14074 (PMC6175012; doi:10.1111/ejn.14074)
Supplement: Supplementary file 1 [file EJN-48-2050-s001.docx]

Changes of motor corticobulbar projections following different lesion types affecting the central nervous system in adult macaque monkeys

Rouiller, Eric M; Fregosi, Michela; Contestabile, Alessandro; Badoud, Simon; Borgognon, Simon; Cottet, Jérôme; Brunet, Jean-François; Bloch, Jocelyne; Schwab, Martin

_____________________________________________________________________________________

| Review timeline: | Submission date: 12.01.18  Editorial Decision: Major Revision  Revision Received:25.04.18  Editorial Decision: Minor Revision  Revision Received: 28.06.18  Accepted: 07.07.18 |  |
| --- | --- | --- |
|  |  |  |

Editor: Helen Barbas

Reviewer 1: Elena Borra

Reviewer 2: Roger Lemon

_____________________________________________________________________________________

1st Editorial Decision	18/02/2018

__________________________________________________________________________________ Dear Prof. Rouiller,

Your manuscript was reviewed by external reviewers as well as by the Section Editor, Dr. Helen Barbas, and ourselves. The Reviewers commented positively on the significance of the topic, and provided a list of thoughtful comments to help clarify and focus your study, and assist the interpretation of the findings. To appeal to a general audience, it is necessary to provide the broad motivation of the study starting with the abstract. A major concern is the lack of direct comparison in the pathway labeling found in intact animals from your previous study, with animals subjected to various lesions in this study. Even though the anatomic pathway data on intact monkeys were recently published, a depiction of these data and direct comparison with the lesioned animals is needed, with the appropriate reference to the published study in the figures and text. In view of the complexity of the methods and small number of animals in each group, it is imperative to elaborate on appropriate controls. For example, the placement of the injections and amount of tracer injected varies, as is clear in the Table and figures. The method of using an internal control to count the axons before termination should be described more fully in this paper as well, because it is critical to interpretation of the findings. Further detail in Methods, in general, is needed, including the welfare status of the animals and precautions taken for the care of the lesioned animals. References to statistical analysis need to be clarified, and the type of statistics used should be described in Methods. The findings and interpretation would also be enriched by description of the behavioral data, and when placed in a physiological context concerning probable mechanism for the observed changes. The Reviewers have provided a list of other comments to help as you revise the manuscript. Finally, it is necessary to go over the manuscript carefully to ensure proper expression (e.g., references to MPTP intoxicated monkeys should be changed to: MPTP lesioned monkeys, or toxin lesioned monkeys; other errors include: Page 3, last paragraph, ‘damages’ should be: damage. Page 4, top, ‘such tremors’, should be: such as tremors; Page 6: ‘were confronted to data obtained’, should be: were compared to data obtained; the list is not exhaustive).

When revising the manuscript, please embolden or underline major changes to the text so they are easily identifiable and DO NOT leave 'track change' formatting marks in your paper. Please ensure that you provide a text and a figure file for the Graphical Abstract (as detailed in the instructions below). When carrying out your revisions please refer to the checklist below and visit the EJN author guidelines at www.ejneuroscience.org

When finalized, please upload your complete revised manuscript onto the website, as a Word file (.doc, or .docx). Please also ensure that a complete set of tables and figures is included as separate files, even if these have not changed from the originals. At this stage it is necessary to provide high resolution figures. Please see important instructions below.

Please go into https://mc.manuscriptcentral.com/ejn - Author Centre - manuscripts with decisions where you will find a 'create a revision' link under 'actions'. We ask that you please indicate the way in which you have responded to the points raised by the Editors and Reviewers in a letter. Please upload this response letter as a separate Word (.doc or PDF) file using the file designation "Authors' Response to Reviewers" when uploading your manuscript files. Please DO NOT submit your revised manuscript as a new one. Also, please note that only the Author who submitted the original version of the manuscript should submit a revised version.

If you are able to respond fully to the points raised, we would be pleased to receive a revision of your paper within 12 weeks.

Thank you for submitting your work to EJN.

Kind regards,

John Foxe & Paul Bolam
co-Editors in Chief, EJN


Reviews:

Reviewer: 1

Comments to the Author
In this manuscript Fregosi and colleagues analyze a series of macaque brains coming from different lesion studies in order to describe the corticoreticular projections originating from area M1 or the PM cortex after a period of recovery in some cases including a specific experimental treatment. The authors compare the results obtained in these cases with those obtained in intact monkeys in which the corticoreticular projections from M1 and PM were analyzed in the same way (Fregosi et al. 2017). The limits of this study, involving a small number of animals for each condition (lesion + treatment), are clearly discussed as well as the more solid results. This manuscript tends to describe in a rigorous way a collection of interesting data on the rearrangement of the corticoreticular projections in various pathological conditions. The exact role of these descending pathways in the recovery after lesions is still largely unknown.

General comments
For interpreting the results of the present study, a comparison with the results described in Fregosi et al. 2017 is crucial. However, only in Figure 3 the data (range and mean) from intact monkeys are represented. Given that there are several combinations of injected area, type of lesion, and treatment, it would be of great help for the reader to more easily access the key results from control monkeys (M1 or PM injections) along the manuscript, in the text and/or figures.

The present study describes the anatomical rearrangement after a recovery period, but the description of the individual level of recovery is completely missing (described in other papers). The first sentence about recovery is in the discussion (page 12, first sentence of the last paragraph). I think that behavioral data relative to the recovery of motor functions should be briefly provided for each subject, in order to better contextualize the anatomical results. Table 1 includes the age (rounded) of each monkey at lesion and at sacrifice, thus it is possible just to approximately calculate only the post-lesion period.

Specific comments
Please indicate (in the methods or in the figure legend) which were the nuclei grouped as “other” and (if possible) the undefined areas. In some case these “regions” include a great percentage of the labeling.
Please describe also in the methods the statistical analysis.
Results. It is impossible to follow the comparison of the regional distribution of the labeling found in the different cases with that of the intact monkeys (for example about the raphe nuclei and the PnO-PnC), as neither in the text nor in the figures these “control” data are summarized and compared.
Page 10 second paragraph: are the authors referring to the projections from both M1 and PM in PD monkeys? It seems that only for M1 this is true.
In figure 1 panel A there are two green regions: a darker one and a lighter one. Do they correspond to the core and the halo of the injection site, respectively, or the lighter region is an artifact? Furthermore, in this figure the hemispheres (especially MK-AC) and the spinal cord sections would be more clearly visible if a bit enlarged.
Supplementary figure 1: it is not clear how the statistical analysis between ipsi- and contralateral boutons was done.
Discussion, page 16: second paragraph, last sentence. This sentence is misleading. It is an increase in the percentage of boutons found in Pn, not in the absolute number, is it correct? And it seems not true for the PM projections but only for the M1 projections.

Reviewer: 2

Comments to the Author
This Ms describes a wide range of studies in which corticobulbar projections from premotor and motor cortex were quantitatively analysed in a group of monkeys subjected to either motor cortex injury, MPTP-parkinsonism or spinal cord injury. Some of the monkeys received putatively therapeutic treatments (anti-NOGO or autologous stem cells). Anatomical studies were carried out 3-8 months after induction of lesions.The main findings are that after motor cortex injury there was a dramatic fall in the number of corticobulbar boutons labelled from premotor cortex and this fall was somewhat lower, but still dramatic, in the anti-NOGO treated animals. This fall also seen in the PD-monkeys, all of whom received ANCE treatment. After a hemisection spinal injury, corticobulbar projections from motor were strongly enhanced, particularly in treated monkeys. Detailed analysis of the laterality of projections and their distributions within the reticular formation are also presented.

GENERAL COMMENT
This study, together with the earlier studies in intact monkeys, provides important new quantitative information about plastic change after lesions, the involvement of brainstem motor pathways and changes in cortical influence over those pathways. To assess their functional importance in recovery from lesions, the changes reported here now need further explanation in terms of the physiological actions on the, for example, cells of origin of the reticulospinal tract. If the corticobulbar projections from e.g. PM, normally exert inhibitory control over the RST, the changes reported here could represent a release mechanism, assisting RST-mediated movements.
DETAILED COMMENTS
1. Some additional information on the welfare status of the monkeys used could be added to the Methods section. Authors should consult the NC3Rs ARRIVE guidelines which apply to submissions to EJN. For example, stating that the monkeys were purpose-bred for research, and details of their social housing could be added.
2. P5 4 lns up ‘….easier access to the muscles targeted for injection’ would be clearer.
3. P9 para 3 ‘….trend towards a greater decrease in the density’
4. P10 para 4 last ln ‘…as was the case in intact monkeys’
5. P12 para 2 ln 7. Is anything known about the CS projections from PM after MCI? Could that be added here?
6. In the Discussion could the authors tackle one question that bothers me. When comparing corticobulbar projections from e.g. PM vs M1, one is comparing the number of projections from a given volume if tissue that is labelled with BDA. From the classical neuroanatomical work, it might be expected that corticobulbar projections from PM are more numerous than from M1, but M1 surely represents a much larger total volume of cortical tissue than PM, so does that difference need to be taken into account when considering the implications of this study?
7. P14 para 2. Would ‘influence’ be better than ‘interfere’?
8. P14 para 3 ln 8. Perhaps ‘impacted directly by the cortical lesion’ (i.e. the lesion did not include PM)?
9. P16 para 4. I think this should read ‘….control of distal muscles allowing CRUDE motor function…Zaaimi et al did not carry out detailed tests of hand function in all the monkeys used in their PT lesion studies, but they did test one, and state ’there was (as expected) no recovery of fine independent finger movements’.

_____________________________________________________________________________________

Authors’ Response	18/02/2018

_____________________________________________________________________________________

Fregosi et al., EJN 2018-01-25255

Reply to reviewers.

Please see below replies and comments in red in the «Decision» e-mail.

Changes in the manuscript itself are underlined.

Manuscript Number:EJN-2018-01-25255 Changes of motor corticobulbar projections following different lesion types affecting the central nervous system in adult macaque monkeys

Dear Prof. Rouiller,

Your manuscript was reviewed by external reviewers as well as by the Section Editor, Dr. Helen Barbas, and ourselves. The Reviewers commented positively on the significance of the topic, and provided a list of thoughtful comments to help clarify and focus your study, and assist the interpretation of the findings. To appeal to a general audience, it is necessary to provide the broad motivation of the study starting with the abstract. Done, a first sentence was added to the abstract in order to introduce the context of rearrangement of neural circuits in relation to a CNS lesion (see revised abstract page 2).

A major concern is the lack of direct comparison in the pathway labeling found in intact animals from your previous study, with animals subjected to various lesions in this study. Even though the anatomic pathway data on intact monkeys were recently published, a depiction of these data and direct comparison with the lesioned animals is needed, with the appropriate reference to the published study in the figures and text. In view of the complexity of the methods and small number of animals in each group, it is imperative to elaborate on appropriate controls. For example, the placement of the injections and amount of tracer injected varies, as is clear in the Table and figures. A whole paragraph was introduced at the beginning of the results section in the revised version, in order to remind the characteristics of the corticobulbar motor projections in intact monkeys originating from distinct motor cortical areas, with focus on M1 and PM. It will therefore be more comfortable for the reader to make the comparison with the cases subjected to lesions and presented here (see pages 9-10).

The method of using an internal control to count the axons before termination should be described more fully in this paper as well, because it is critical to interpretation of the findings. The procedure of normalization has been described in more detail, especially how the number of CS axons used as a reference was established (see page 8).

Further detail in Methods, in general, is needed, including the welfare status of the animals and precautions taken for the care of the lesioned animals. References to statistical analysis need to be clarified, and the type of statistics used should be described in Methods. All these aspects have been introduced in the revised versions of the Methods (see page 5, page 9 for statistics).

Page 1 of 57 European Journal of Neuroscience

For Peer Review

The findings and interpretation would also be enriched by description of the behavioral data, and when placed in a physiological context concerning probable mechanism for the observed changes. The revised version comprises now a new Table (Table 2), giving for each animal data about functional recovery from the corresponding lesion (new Table 2 introduced in the text on page 9).

The Reviewers have provided a list of other comments to help as you revise the manuscript. Finally, it is necessary to go over the manuscript carefully to ensure proper expression (e.g., references to MPTP intoxicated monkeys should be changed to: MPTP lesioned monkeys, or toxin lesioned monkeys; other errors include: Page 3, last paragraph, ‘damages’ should be: damage. Page 4, top, ‘such tremors’, should be: such as tremors; Page 6: ‘were confronted to data obtained’, should be: were compared to data obtained; the list is not exhaustive). All these modifications have been inserted in the revised version. Furthermore, the revised manuscript was checked for grammar and syntax by a native English speaking colleague.

Two very recent and pertinent references were introduced in the revised version: Asboth et al., 2018 and Zaaimi et al., 2018 (see discussion).

When revising the manuscript, please embolden or underline major changes to the text so they are easily identifiable and DO NOT leave 'track change' formatting marks in your paper. Please ensure that you provide a text and a figure file for the Graphical Abstract (as detailed in the instructions below). When carrying out your revisions please refer to the checklist below and visit the EJN author guidelines at www.ejneuroscience.org

When finalized, please upload your complete revised manuscript onto the website, as a Word file (.doc, or .docx). Please also ensure that a complete set of tables and figures is included as separate files, even if these have not changed from the originals. At this stage, it is necessary to provide high resolution figures. Please see important instructions below.

Please go into https://mc.manuscriptcentral.com/ejn - Author Centre - manuscripts with decisions where you will find a 'create a revision' link under 'actions'. We ask that you please indicate the way in which you have responded to the points raised by the Editors and Reviewers in a letter. Please upload this response letter as a separate Word (.doc or PDF) file using the file designation "Authors' Response to Reviewers" when uploading your manuscript files. Please DO NOT submit your revised manuscript as a new one. Also, please note that only the Author who submitted the original version of the manuscript should submit a revised version.

If you are able to respond fully to the points raised, we would be pleased to receive a revision of your paper within 12 weeks.

Thank you for submitting your work to EJN.

Kind regards,

John Foxe & Paul Bolam co-Editors in Chief, EJN

Reviews:

Reviewer: 1

Comments to the Author

Page 2 of 57European Journal of Neuroscience

For Peer Review

In this manuscript Fregosi and colleagues analyze a series of macaque brains coming from different lesion studies in order to describe the corticoreticular projections originating from area M1 or the PM cortex after a period of recovery in some cases including a specific experimental treatment. The authors compare the results obtained in these cases with those obtained in intact monkeys in which the corticoreticular projections from M1 and PM were analyzed in the same way (Fregosi et al. 2017). The limits of this study, involving a small number of animals for each condition (lesion + treatment), are clearly discussed as well as the more solid results. This manuscript tends to describe in a rigorous way a collection of interesting data on the rearrangement of the corticoreticular projections in various pathological conditions. The exact role of these descending pathways in the recovery after lesions is still largely unknown.

General comments For interpreting the results of the present study, a comparison with the results described in Fregosi et al. 2017 is crucial. However, only in Figure 3 the data (range and mean) from intact monkeys are represented. Given that there are several combinations of injected area, type of lesion, and treatment, it would be of great help for the reader to more easily access the key results from control monkeys (M1 or PM injections) along the manuscript, in the text and/or figures. As mentioned above, a new paragraph in the Results section now summarizes the main characteristics of the motor corticobulbar (corticoreticular) projections in intact monkeys, for an easier comparison with the lesioned cases (pages 9-10). Furthermore, the newly introduced “Graphical Abstract” emphasizes also the properties of the normal projection.

The present study describes the anatomical rearrangement after a recovery period, but the description of the individual level of recovery is completely missing (described in other papers). The first sentence about recovery is in the discussion (page 12, first sentence of the last paragraph). I think that behavioral data relative to the recovery of motor functions should be briefly provided for each subject, in order to better contextualize the anatomical results. Table 1 includes the age (rounded) of each monkey at lesion and at sacrifice, thus it is possible just to approximately calculate only the post-lesion period. See above, newly introduced Table 2 in the revised version (see page 9).

Specific comments Please indicate (in the methods or in the figure legend) which were the nuclei grouped as “other” and (if possible) the undefined areas. In some case these “regions” include a great percentage of the labeling. See legend of Supplementary Figure 1 for “Others”. Undefined are for boutons which were located at positions which could not be attributed to a well defined nucleus, at least in our histological material.

Please describe also in the methods the statistical analysis. Done (see page 9).

Results. It is impossible to follow the comparison of the regional distribution of the labeling found in the different cases with that of the intact monkeys (for example about the raphe nuclei and the PnOPnC), as neither in the text nor in the figures these “control” data are summarized and compared. See above, reminder of the normal projections in intact monkeys (see pages 9-10).

Page 10 second paragraph: are the authors referring to the projections from both M1 and PM in PD monkeys? It seems that only for M1 this is true. Correct, modified accordingly (see page 11).

In figure 1 panel A there are two green regions: a darker one and a lighter one. Do they correspond to the core and the halo of the injection site, respectively, or the lighter region is an artifact? Modified accordingly in the legend of Figure 1.

Page 3 of 57 European Journal of Neuroscience

For Peer Review

Furthermore, in this figure the hemispheres (especially MK-AC) and the spinal cord sections would be more clearly visible if a bit enlarged. Figure 1 modified accordingly. Supplementary figure 1: it is not clear how the statistical analysis between ipsi- and contralateral boutons was done. Now described in the methods (see page 9).

Discussion, page 16: second paragraph, last sentence. This sentence is misleading. It is an increase in the percentage of boutons found in Pn, not in the absolute number, is it correct? And it seems not true for the PM projections but only for the M1 projections. Modified accordingly (see page 17).

Reviewer: 2

Comments to the Author This Ms describes a wide range of studies in which corticobulbar projections from premotor and motor cortex were quantitatively analysed in a group of monkeys subjected to either motor cortex injury, MPTP-parkinsonism or spinal cord injury. Some of the monkeys received putatively therapeutic treatments (anti-NOGO or autologous stem cells). Anatomical studies were carried out 38 months after induction of lesions. The main findings are that after motor cortex injury there was a dramatic fall in the number of corticobulbar boutons labelled from premotor cortex and this fall was somewhat lower, but still dramatic, in the anti-NOGO treated animals. This fall also seen in the PDmonkeys, all of whom received ANCE treatment. After a hemisection spinal injury, corticobulbar projections from motor were strongly enhanced, particularly in treated monkeys. Detailed analysis of the laterality of projections and their distributions within the reticular formation are also presented.

GENERAL COMMENT This study, together with the earlier studies in intact monkeys, provides important new quantitative information about plastic change after lesions, the involvement of brainstem motor pathways and changes in cortical influence over those pathways. To assess their functional importance in recovery from lesions, the changes reported here now need further explanation in terms of the physiological actions on the, for example, cells of origin of the reticulospinal tract. If the corticobulbar projections from e.g. PM, normally exert inhibitory control over the RST, the changes reported here could represent a release mechanism, assisting RST-mediated movements. Such “physiological action” has been introduced in the revised discussion (see page 16). See also very last part of the discussion.

DETAILED COMMENTS 1. Some additional information on the welfare status of the monkeys used could be added to the Methods section. Authors should consult the NC3Rs ARRIVE guidelines which apply to submissions to EJN. For example, stating that the monkeys were purpose-bred for research, and details of their social housing could be added. Information added in the revised Methods (see page 5).

2. P5 4 lns up ‘….easier access to the muscles targeted for injection’ would be clearer. Done (Page 5). 3. P9 para 3 ‘….trend towards a greater decrease in the density’. Done (Page 10). 4. P10 para 4 last ln ‘…as was the case in intact monkeys’. Done (Page 12). 5. P12 para 2 ln 7. Is anything known about the CS projections from PM after MCI? Could that be added here?

Page 4 of 57European Journal of Neuroscience

For Peer Review

We have a very large material about the CS projection from PM, both in intact monkeys and lesioned monkeys (different types of lesions). This represent an enormous material presently under analysis and which is a separate project in itself (the topic of a future Ph.D. thesis). For this reason, it cannot be shortly reported here.

6. In the Discussion could the authors tackle one question that bothers me. When comparing corticobulbar projections from e.g. PM vs M1, one is comparing the number of projections from a given volume if tissue that is labelled with BDA. From the classical neuroanatomical work, it might be expected that corticobulbar projections from PM are more numerous than from M1, but M1 surely represents a much larger total volume of cortical tissue than PM, so does that difference need to be taken into account when considering the implications of this study?

M1 may be larger than PM (PMd and PMv), but probably not "much" larger. This is difficult to evaluate how much, because borders between these motor areas are not strict, but correspond to a functional transition, not appearing on histological sections. In other words, it would be difficult to compute a reliable “total” volume for M1 and for PM. For the latter, which subdivisions to include (include F7 or not?). The corticobulbar projections from PM and M1 published in Fregosi et al., 2017 are based on cases in which a fairly large part of PM was injected with BDA and less so in M1 (mostly hand/arm areas). This is what is visible on their Fig. 2, although the size of the BDA injections in M1 is underestimated in this representation, as we do not see the whole portion of M1 injected in the rostral bank of the central sulcus (new M1), not apparent on this surface view. Actually, the volumes of BDA injected were clearly larger in the M1 cases than in the PM cases (3 times more in two M1 monkeys, slightly more in the third one). As a result, overall the injection sites in M1 are actually bigger than in PM. This difference then may counterbalance, at least to some extent, the fact that M1 is overall larger than PM. Then, in the intact monkeys (Fregosi et al., 2017), we did not dramatically under-sampled M1 as compared to PM, with respect to their total volume. In the present study, in the 2 groups "MCI" and "SCI" (Table 1), the volumes of BDA injected in PM and M1, respectively, are comparable to those injected in PM and in M1 in intact monkeys (in M1 comparable to the 2 large injections). Only in the "MPTP" group, the volume of BDA injected in M1 was smaller than in the intact monkeys, namely comparable to the volume injected in PM. This may actually explain the modest decrease of the corticobulbar projection from M1 in the PD monkeys. With respect to a "theoretical" total corticobulbar projection, what would be its amount if we would consider the entire M1 (including face, trunk, hindlimb areas)? This is not possible to assess because there is no evidence that the corticobulbar projection is homogeneous somatotopically: is the density of the corticobulbar projection the same for the face, hand and leg areas? Because of this uncertainty, any extrapolation would remain very speculative. Another issue is related to the number of CS axons. In the frontal lobe, it is known that about 50% of them originate from M1 and the other 50% from PM, SMA and CMA. One can approximate their proportion in PM to be about 10-15%. From Keizer and Kuypers (1989), one third of corticobulbar projections are collateral of CS axons (the other two thirds separate projections). Based on that (CS projection), if one injects BDA in a given volume of cortex (in M1 or in PM) and hypothesizes that M1 is 3x to 5x bigger than PM, one may expect a fairly comparable number of collateralized corticobulbar projections (as there is 3 to 5 times more CS neurons in M1 than in PM). In most of our cases, as the volume of BDA injected was clearly bigger in M1 than in PM (except the MPTP cases), this represented a measure to minimize the bias towards PM due to its small size as compared to M1. We have addressed this issue in the discussion (as requested by the reviewer: see pages 14-15), however in a shorter version of the elements given above.

7. P14 para 2. Would ‘influence’ be better than ‘interfere’? Done (Page 16).

Page 5 of 57 European Journal of Neuroscience

For Peer Review

8. P14 para 3 ln 8. Perhaps ‘impacted directly by the cortical lesion’ (i.e. the lesion did not include PM)? Done (Page 16). 9. P16 para 4. I think this should read ‘….control of distal muscles allowing CRUDE motor function…Zaaimi et al did not carry out detailed tests of hand function in all the monkeys used in their PT lesion studies, but they did test one, and state ’there was (as expected) no recovery of fine independent finger movements’. Done (Page 18).

_____________________________________________________________________________________

2nd Editorial Decision	07/06/2018

_____________________________________________________________________________________

Dear Prof. Rouiller,

Your revised manuscript has been re-evaluated by external reviewers as well as by the Section Editor, Dr. Helen Barbas and ourselves.  As you can see, the Reviewers appreciated your effort in responding to the points raised and we are pleased to inform you that it will be accepted for publication in EJN after you have dealt with a few minor points.

- Clarify what is meant by adhering to the NC3Rs initiative.
- Please supply a data statement.
- Please make the corrections listed below:
‘e.g.’ change to: e.g.,
Page 9: ‘Brief reminder..’, change to: Brief summary
Page 10: ‘First of all..’, change to: First,
Page 10: ‘Secondly,..’, change to: Second,
Page 10: ‘Thirdly,..’, change to: Third,
Please also change these terms when used in the Discussion.
Page 14: ‘..much bigger,..’, change to: much larger
Page 15: ‘..cannot exclude then..’, change to: cannot exclude the possibility
Page 16: ‘In other words and in case the corticobulbar projection would normally exert an inhibitory effect, then a decrease of the density of the corticobulbar projection after MCI or MPTP lesion would lead to a disinhibition of the cells of origin of the reticulospinal system.’ This sentence is confusing: See if this is what you mean: In other words, if the corticobulbar projection normally exerts an inhibitory effect, then a decrease in its density after MCI or MPTP lesion would lead to disinhibition of the cells of origin of the reticulospinal system.

Once done we will send it off to the publishers.

When revising the manuscript, please embolden or underline major changes to the text so they are easily identifiable and DO NOT leave 'track change' formatting marks in your paper.  At this stage, please provide text and a figure file for the Graphical Abstract.  When carrying out your revisions please refer to the checklist below and visit the EJN author guidelines at www.ejneuroscience.org

When finalised, please upload your complete revised manuscript onto the website as a Word (.doc or .docx), or .rtf file.  Please also ensure that a complete set of tables and figures is included as separate files, even if these have not changed from the originals.  At this stage it is necessary to provide high resolution figures.  Please see important instructions below.

Please go into https://mc.manuscriptcentral.com/ejn - Author Centre - manuscripts with decisions where you will find a 'create a revision' link under 'actions'. We ask that you please indicate the way in which you have responded to the points raised by the Editors and Reviewers in a letter. Please upload this response letter as a separate Word (.doc) file using the file designation "Authors' Response to Reviewers" when uploading your manuscript files. Please DO NOT submit your revised manuscript as a new one.  Also, please note that only the  Author who submitted the original version of the manuscript should submit a revised version.

If you are able to respond fully to the points raised, we shall be pleased to receive a revision of your paper within 30 days.

Thank you for submitting your work to EJN.

Best wishes,

Paul Bolam & John Foxe
co-Editors in Chief, EJN

Reviews:

Reviewer: 2

Comments to the Author
I thank the authors for responding satisfactorily to all my comments.

On last point, could the authors define more clearly what they mean on p5 by  "The authors adhere to the NC3Rs initiative"?

Reviewer: 1

Comments to the Author
The authors replied to all my comments adequately. I would have expected a more extensive revision, but I think that now the manuscript is acceptable for publication.

_____________________________________________________________________________________

Authors’ Response	07/06/2018

_____________________________________________________________________________________

Fregosi et al., EJN 2018-01-25255.R1

Reply to reviewers.

Please see below replies and comments in red in the «Decision» e-mail.

Changes in the manuscript itself are underlined.

07-Jun-2018

Manuscript Number:EJN-2018-01-25255.R1 Changes of motor corticobulbar projections following different lesion types affecting the central nervous system in adult macaque monkeys

Dear Prof. Rouiller,

Your revised manuscript has been re-evaluated by external reviewers as well as by the Section Editor, Dr. Helen Barbas and ourselves. As you can see, the Reviewers appreciated your effort in responding to the points raised and we are pleased to inform you that it will be accepted for publication in EJN after you have dealt with a few minor points.

- Clarify what is meant by adhering to the NC3Rs initiative. This has been addressed in the R2 revision (see page 5, underlined sentence). The signification of the 3Rs has been inserted, as well as the web site address of the nc3rs initiative. - Please supply a data statement. A data statement about accessibility has been added in the R2 revision (see page 20). - Please make the corrections listed below: ‘e.g.’ change to: e.g., Page 9: ‘Brief reminder..’, change to: Brief summary Page 10: ‘First of all..’, change to: First, Page 10: ‘Secondly,..’, change to: Second, Page 10: ‘Thirdly,..’, change to: Third, Please also change these terms when used in the Discussion. Page 14: ‘..much bigger,..’, change to: much larger Page 15: ‘..cannot exclude then..’, change to: cannot exclude the possibility Page 16: ‘In other words and in case the corticobulbar projection would normally exert an inhibitory effect, then a decrease of the density of the corticobulbar projection after MCI or MPTP lesion would lead to a disinhibition of the cells of origin of the reticulospinal system.’ This sentence is confusing: See if this is what you mean: In other words, if the corticobulbar projection normally exerts an inhibitory effect, then a decrease in its density after MCI or MPTP lesion would lead to disinhibition of the cells of origin of the reticulospinal system. All these corrections were made accordingly (see underlined words/sentences).

Once done we will send it off to the publishers.

When revising the manuscript, please embolden or underline major changes to the text so they are easily identifiable and DO NOT leave 'track change' formatting marks in your paper. At this stage, please provide text and a figure file for the Graphical Abstract. When carrying out your revisions please refer to the checklist below and visit the EJN author guidelines at www.ejneuroscience.org

When finalised, please upload your complete revised manuscript onto the website as a Word (.doc or .docx), or .rtf file. Please also ensure that a complete set of tables and figures is included as separate files, even if these have not changed from the originals. At this stage it is necessary to provide high resolution figures. Please see important instructions below.

Please go into https://mc.manuscriptcentral.com/ejn - Author Centre - manuscripts with decisions where you will find a 'create a revision' link under 'actions'. We ask that you please indicate the way in which you have responded to the points raised by the Editors and Reviewers in a letter. Please upload this response letter as a separate Word (.doc) file using the file designation "Authors' Response to Reviewers" when uploading your manuscript files. Please DO NOT submit your revised manuscript as a new one. Also, please note that only the Author who submitted the original version of the manuscript should submit a revised version.

If you are able to respond fully to the points raised, we shall be pleased to receive a revision of your paper within 30 days.

Thank you for submitting your work to EJN.

Best wishes,

Paul Bolam & John Foxe co-Editors in Chief, EJN

Reviews:

Reviewer: 2

Comments to the Author I thank the authors for responding satisfactorily to all my comments.

On last point, could the authors define more clearly what they mean on p5 by "The authors adhere to the NC3Rs initiative"? See above.

Reviewer: 1

Comments to the Author The authors replied to all my comments adequately. I would have expected a more extensive revision, but I think that now the manuscript is acceptable for publication.
